# Autophagy in Spinocerebellar ataxia type 2, a dysregulated pathway, and a target for therapy

**DOI:** 10.1038/s41419-021-04404-1

**Published:** 2021-11-29

**Authors:** Adriana Marcelo, Inês T. Afonso, Ricardo Afonso-Reis, David V. C. Brito, Rafael G. Costa, Ana Rosa, João Alves-Cruzeiro, Benedita Ferreira, Carina Henriques, Rui J. Nobre, Carlos A. Matos, Luís Pereira de Almeida, Clévio Nóbrega

**Affiliations:** 1 ABC-RI, Algarve Biomedical Center Research Institute, Algarve Biomedical Center, Faro, Portugal; 2 PhD Program in Biomedical Sciences, Faculdade de Medicina e Ciências Biomédicas, Universidade do Algarve, Faro, Portugal; 3 Center for Neuroscience and Cell Biology (CNC), University of Coimbra, Coimbra, Portugal; 4 Faculty of Medicine and Biomedical Sciences, University of Algarve, Faro, Portugal; 5 Faculty of Pharmacy, University of Coimbra, Coimbra, Portugal; 6 Champalimaud Research Program, Champalimaud Center for the Unknown, Lisbon, Portugal

**Keywords:** Diseases of the nervous system, Molecular neuroscience

## Abstract

Spinocerebellar ataxia type 2 (SCA2) is an incurable and genetic neurodegenerative disorder. The disease is characterized by progressive degeneration of several brain regions, resulting in severe motor and non-motor clinical manifestations. The mutation causing SCA2 disease is an abnormal expansion of CAG trinucleotide repeats in the *ATXN2* gene, leading to a toxic expanded polyglutamine segment in the translated ataxin-2 protein. While the genetic cause is well established, the exact mechanisms behind neuronal death induced by mutant ataxin-2 are not yet completely understood. Thus, the goal of this study is to investigate the role of autophagy in SCA2 pathogenesis and investigate its suitability as a target for therapeutic intervention. For that, we developed and characterized a new striatal lentiviral mouse model that resembled several neuropathological hallmarks observed in SCA2 disease, including formation of aggregates, neuronal marker loss, cell death and neuroinflammation. In this new model, we analyzed autophagic markers, which were also analyzed in a SCA2 cellular model and in human post-mortem brain samples. Our results showed altered levels of SQSTM1 and LC3B in cells and tissues expressing mutant ataxin-2. Moreover, an abnormal accumulation of these markers was detected in SCA2 patients’ striatum and cerebellum. Importantly, the molecular activation of autophagy, using the compound cordycepin, mitigated the phenotypic alterations observed in disease models. Overall, our study suggests an important role for autophagy in the context of SCA2 pathology, proposing that targeting this pathway could be a potential target to treat SCA2 patients.

## Introduction

Spinocerebellar ataxia type 2 (SCA2) is a rare and fatal dominantly-inherited polyglutamine (polyQ) disease [1, 2], being caused by abnormal repeats of the triplet CAG in the *ATXN2* gene, resulting in an expanded polyQ chain in the ataxin-2 protein [3, 4]. In healthy individuals, the protein carries between 13–31 glutamines (Q), while repeats above that threshold lead to disease development [5]. The mutation results in progressive and widespread degeneration of different neuronal populations, including the cerebellum, pons, basal ganglia, thalamus and midbrain [6]. The clinical manifestations include motor dysfunctions such as progressive gait ataxia, dysarthria, postural tremors and bradykinesia, as well as visual impairments and cognitive/psychiatric symptoms [7, 8]. Currently, SCA2 patients rely only on pharmacological compounds to relief symptomatology.

Understanding the molecular mechanisms underlying SCA2 pathogenesis is essential to find new targets and an efficient therapy for this incurable disorder. The development and characterization of several SCA2 cellular and animal models, already provided some hints in the events that lead to neuronal death. The disease-causing polyQ expansion induces conformational alterations in the native ataxin-2 structure, resulting in formation of insoluble fibrillar bodies that accumulate as aggregates inside neuronal cells [9, 10]. Although the role of aggregation in SCA2 pathogenesis remains unresolved, analysis of patients’ post-mortem brain samples revealed a positive correlation between the presence of mutant ataxin-2 inclusions and the severity/extension of neuronal degeneration [11]. Moreover, processes such as oxidative stress, disruption of cell signaling and disturbance of calcium homeostasis have been shown to be involved in SCA2 pathogenesis [12]. Despite several advances, the exact mechanisms through each mutant ataxin-2 leads to death of neurons are still controversial and need to be further elucidated.

As mutant polyQ proteins form large multi-protein inclusions, protein degradation systems are essential to maintain cellular homeostasis. An important protein clearance mechanism is autophagy, a key pathway implicated in other polyQ diseases pathogenesis [13]. Autophagic markers, such as LC3B (microtubule-associated protein 1 light-chain 3B) or SQSTM1 (sequestosome 1), have been observed in polyQ pathological aggregates suggesting autophagy impairment [14, 15]. In line with this, we previously demonstrated that pharmacologic activation of autophagy, using the compound cordycepin, reduced the neuropathological and motor deficits in mouse models of Machado-Joseph disease (MJD) (ref. [16]).

In SCA2, the analysis of patients’ mRNA levels revealed increased levels of the autophagic protein *WDFY3* (WD repeat and FYVE domain containing 3) (ref. [17]). Also, STAU1 (staufen 1) protein was found to be augmented in SCA2 patients-derived fibroblasts due to autophagy dysfunction [18]. However, there is a lack of studies investigating the presence of autophagy markers in the context of SCA2, or in the study of autophagy activation as a therapeutic strategy. Therefore, in this work we aimed at evaluating the autophagic process in a new mouse model of SCA2, as well as cellular models and patients’ brain tissues. We found that SQSTM1 and LC3B levels are altered in SCA2, suggesting a dysregulation of autophagy pathway. Importantly, molecular activation of autophagy, through cordycepin, reduced mutant ataxin-2 aggregation in the SCA2 lentiviral mouse model.

## Material and Methods

### Animals

Adult C57BL/6 mice, breed in the animal house facility of the Algarve Biomedical Center Research Institute of the University of Algarve, were used in this work. All animals were housed in a temperature-controlled room on a 12h light–12 h dark cycle. Food and water were provided ad libitum. The number of animals used in each experiment was based in previous studies and in the recommendation to reduce the number of animals used in experimentation. The experiments were carried out in accordance with the European Community directive (86/609/EEC) for the care and use of laboratory animals. The researchers received certified training (FELASA course) and approval to perform the experiments from the Portuguese authorities (Direcção Geral de Alimentação e Veterinária) in the project Neuropath (421/2019).

### Lentiviral vectors

Lentiviral particles (LVs) encoding for human full-length ataxin-2 with either normal polyQ segment (23Q – Atx2WT) or mutant polyQ segment (80Q or 104Q – Atx2MUT) under the control of the mouse phosphoglycerate kinase 1 (PGK) promoter were produced in HEK (human embryonic kidney) 293T cells using a four-plasmid system, described elsewhere [19] and quantified by p24 antigen ELISA (RETROtek, Gentaur, France) by ViraVector facility, University of Coimbra. Additionally, the lentiviral vectors titer was quantified using the Lentivirus qPCR Titer Kit (Applied Biological Materials Inc, Vancouver, Canada), allowing the determination of the virus titer in infectious units per ml (IU/ml).

### Striatal lentiviral mouse model

To develop the SCA2 lentiviral mice, concentrated LVs viral stocks encoding for Atx2WT or Atx2MUT were thawed on ice and homogenized. Adult (8/10-week-old) C57BL/6 mice (both males and females) were anesthetized through intraperitoneal injection (IP) of a mixture of ketamine (75mg/kg, Nimatek, Dechra) with medetomidine (0.75mg/kg, DOMTOR®, Esteve). LVs were administered through a stereotaxic surgery into the left (Atx2WT) or the right (Atx2MUT) hemisphere of the striatum according to the following brain coordinates relative to bregma: antero-posterior (+0.6mm); lateral (±1.8mm); ventral (−3.3 mm). A concentration of 400.000ng of p24 antigen (2E10 IU/µl) in 2 µl of LVs were injected at a rate of 0.25 µl/min, by means of an automatic injector (Stoelting Co., Wood Dale, IL, USA), into mice striatum using a 34-gauge blunt-tip needle linked to a Hamilton syringe (Hamilton, Reno, NV, USA). Four, eight and twelve weeks after surgery, mice were sacrificed for posterior analysis.

### Tissue preparation

For all immunohistochemical assays, animals received an anesthesia (mixture described above) overdose, followed by a transcardial perfusion with 4% paraformaldehyde (PFA) solution SigmaAldrich). Upon removal, brains were left 24h in 4% PFA solution, dehydrated in a 30% sucrose/0.1M phosphate buffer solution (PBS) for 48h and cryoprotected at −80 °C degrees. Posteriorly, coronal brain slices of 20 µm thickness were obtained using a cryostat (Cryostar NX50, ThermoFisher Scientific) and stored in freefloating PBS with 0.05 µm sodium azide solution at 4 °C. For western blotting procedures, animals received an anesthesia overdose, followed by cervical dislocation. Brains were removed and striatal punches, using a Harris Core pen with 2.5 mm diameter (Ted Pella Inc., Redding, California, USA), were collected and stored at −80 °C until subsequent processing.

### Behavioral analysis

For evaluation of exploratory behavior, mice stereotaxically injected with LVs, encoding for either Atx2WT or Atx2MUT in the right striatum hemisphere, were subjected to open field at 4- and 8-weeks post-surgery. Mice were placed in an activity cage (Panlab, Barcelona, Spain) with 50x50cm arena and 50cm-high walls for 40min and movement activity was recorded using the Anti-Track System (Panlab, Barcelona, Spain). The collected data was blindly analyzed for the last 30min of activity. Experimental tests were performed in the same room, with lights turned off and after 60 min minimum of space acclimatization.

### Neuroblastoma cell culture and transfection

Mouse neuroblastoma line-derived cells (Neuro-2A), obtained from the American Type Culture Collection cell biology bank (CCL-131), were cultured in Dulbecco’s modified Eagle’s medium (DMEM) supplemented with 10% fetal bovine serum (FBS), 100 U/ml penicillin and 100 mg/ml streptomycin (Gibco) at 37 °C in 5% CO2/air atmosphere. These cells are routinely tested for mycoplasma contamination. For transfection assays, complexes of 3 µl PEI solution (polyethylenimine 1mg/ml, Tebu-bio) with 500ng of DNA plasmids were formed in 10 µl DMEM without supplementation and added to cells previously cultured in 12-well plates for 24h. Transfection reagents were left in cells for a 48h-period incubation, following cells harvest for immunocytochemistry or western blot assays. The following DNA constructs were used: human full-length ataxin-2 with normal polyQ segment (23Q – Atx2WT) and mutant polyQ segment (80Q – Atx2MUT); human full-length ataxin-2 with an EGFP (enhanced green fluorescent protein) tag carrying normal polyQ segment (22Q – Atx2-Q22) or mutant polyQ segment (58Q – Atx2-Q58 or 104Q – Atx2-Q104) (ref. [20]); ataxin-2 truncated form with an EGFP tag carrying normal polyQ segment (22Q – Atx2-Q22T) or mutant polyQ segment (58Q – Atx2-Q58T); ptfLC3-RFP-GFP (Addgene #21074) (ref. [21]). For chloroquine (ChQ) experiments, cells were treated 6h before harvest with 100 µM of 1mg/ml ChQ (Sigma) solution in DMSO. Starvation condition was induced by replacing cells culture medium with Hanks’ Balanced Salt Solution (HBSS 1X) (Gibco) supplemented with 1.8g/L of glucose 6h before harvest.

### Cordycepin treatment – in vitro and in vivo studies

For in vitro studies, Neuro-2A cells transfected with EGFP Atx2-Q104 construct or ptfLC3-RFP-GFP were treated with 20 μM cordycepin (3’-deoxyadenosine, 300mg/ml in DMSO, TargetMol) for 48h and harvested for direct fluorescence analysis.

For in vivo experiments, Adult (8/10-week-old) C57BL/6 mice (both males and females) were stereotaxically injected with LVs, encoding for Atx2MUT in the right striatum hemisphere. For the long-term treatment, cordycepin administration began one week after surgery. Treated mice were administered an IP injection of 20mg/Kg of cordycepin in DMSO-NaCl 0.1% solution, while control group received an IP injection of the vehicle (NaCl 0.1%), 2x per week for five weeks, 3x per week during following four weeks and 5x per week during final three weeks (the animals were randomly distributed in each group after the stereotaxic injection). The concentrations and timepoints were defined considering our previous study using cordycepin in in vivo experiments [16]. After final treatment week, mice were sacrificed for analysis. In the short-term treatment, the animals received either an IP injection of 20 mg/Kg of cordycepin in DMSO-NaCl 0.1% solution or an IP injection of the vehicle (NaCl 0.1%) 5x per week for 4 weeks. After the final treatment week, mice were sacrificed for molecular analysis.

### Human post-mortem brain tissue

Post-mortem striatum and cerebellum brain tissue from two clinically and genetically SCA2 confirmed patients were obtained from the NIH NeuroBioBank (USA). Control striatum and cerebellum tissues from healthy individuals, without neurological conditions diagnosed, were obtained from NIH NeuroBioBank (USA). Tissues preserved in 4% PFA solution, were dehydrated in a 30% sucrose/PBS for 48h, cryoprotected at −80 °C degrees, dissected in 40 µm slices using a cryostat (Cryostar NX50, ThermoFisher Scientific) and stored in free floating PBS/sodium azide solution at 4 °C.

### Immunochemical procedures

#### Immunocytochemistry

Immunocytochemistry procedure was performed based on protocols described elsewhere [22, 23]. Briefly, for cells harvest, we washed cultures with PBS and fixed it with 4% PFA for 20 min. Cells were permeabilized with 1% Triton™ X-100 (Sigma) and blocked using 3% Bovine Serum Albumin (Nzytech) in PBS. Incubation with primary antibodies was done overnight at 4 °C. After PBS washes for antibodies removal, cells were incubated in secondary antibodies for 2h at room temperature (RT). Finally, cells were washed with PBS and coverslips were mounted on microscope slides using Fluoromount-G™ medium with DAPI (4′,6′-Diamidino-2′-phenylindole) (ThermoFisher Scientific). Images were acquired with 40x objective in a Andor benchtop spinning disk confocal instrument (Oxford Instruments, UK) and with a 63x and 100x objectives in a Zeiss LSM710 confocal microscope.

#### Immunohistochemistry – free floating incubation

Immunohistochemistry assays were performed based on protocols described elsewhere [14, 24, 25]. For visible microscopy analysis, mouse and human brain slices were incubated in PBS/0.1% diphenylhydrazine for 30 min at 37 °C. A subsequent step of incubation in Tris-buffered pH = 9 antigen retrieval solution for 30min at 95 °C was performed for human sections. Then, all sections were left in PBS/10% normal goat serum (Gibco)/0.1% Triton X blocking solution for 1h at RT, followed by overnight incubation with primary antibodies at 4 °C. Posteriorly, slices were incubated in biotinylated secondary antibodies (1:200, Vector Laboratories) for 2h at RT and then in a reaction with the Vectastain elite avidin-biotin-peroxidase kit and by 3,3′-diaminobenzidine substrate (both from Vector Laboratories). After, sections were assembled over microscope slides, dehydrated in increasing degree ethanol solutions (75, 96 and 100%) and xylene, and finally coverslipped using mounting medium Eukitt (O. Kindler GmbH & CO, Freiburg, Germany). For fluorescence-labeling procedures, sections were incubated in the same blocking solution, followed primary and secondary antibodies incubation as described above. After antibodies probing, slices were mounted in microscope slides and coverslipped using the Fluoromount-G™ medium with DAPI. Images were acquired with 20x objective in a Zeiss Axio Imager Z2 and Axio Scan.Z1 Slide Scanner microscopes.

#### Immunochemical antibodies

For immunochemical procedures, the following primary antibodies were used: mouse anti-ataxin-2 (1:1000, ref. 611378, BD Biosciences); rabbit anti-DARP-32 (1:1000, ref. AB10518, Merck Millipore); mouse anti-Iba1/AIF1 (1:1000, ref. MABN92, Merck Millipore); mouse anti-GFAP (1:1000, ref. 644702, BioLegend); rabbit anti-LC3B (1:1000, ref. NB100-2220, Novus); rabbit anti-SQSTM1 (1:1000, ref. 5114S, Cell Signaling); rabbit anti-Cleaved Caspase-3 (Asp175) (1:1000, ref. 9661, Cell Signaling); rabbit anti-Cleaved Caspase-3 (Asp175) (1:1000, ref. 9661, Cell Signaling); rabbit anti-GFAP (1:500, ref. Z0334, Dako, Agilent); rabbit anti-Iba1 (1:1000, ref. 019-19741, FUJIFILM Wako Pure Chemical Corporation) and rabbit anti-NeuN (1:1000, ref. ABN78, Millipore).

### Cresyl violet staining

Coronal mouse brain slices were displayed onto microscope slides, followed by cresyl violet solution staining and ethanol (75, 96 and 100% solutions) and xylene dehydration. Eukitt mounting medium was then used to mount coverslips. Images were acquired with 20x objective in a Zeiss Axio Imager Z2.

#### Immunochemistry quantitative analysis

For quantification of ataxin-2 aggregates and DARPP-32 staining loss, 18 coronal sections per animal were analyzed in ZEN lite software (Zeiss), so that a complete rostrocaudal picture of the striatum was obtained (14-16 transduced sections). Ataxin-2 inclusions were manually counted in all animals in a blind manner. DARPP-32 neuronal lesion area was manually measured for all animals, allowing quantification of depleted volume according to the formula: volume = d*(a1 + a2 + a3), where d is the distance between serial sections (200 μm) and a1 + a2 + a3 are depleted areas for each individual section.

#### Cleaved caspase-3 quantitative analysis

The cleaved caspase-3 puncta were assessed by scanning 14-16 sections of each animal covering the entire transduced striatum using a 20x objective in an Axio Scan.Z1 Slide Scanner microscope. The total number of cleaved caspase-3 puncta was calculated automatically in a blind manner using ImageJ (NIH, USA), as well as the number of ataxin-2 transduced cells in those sections [23]. Data were expressed as the ratio of the total number of cleaved caspase-3 puncta to the total number of ataxin-2 transduced cells (cordycepin versus control group), or as the ratio of the total number of cleaved caspase-3 puncta to the total number of ataxin-2 transduced cells normalized for the control Atx2WT hemisphere (lentiviral model).

#### Neuronal density quantitative analysis

The total number of ataxin-2 transduced cells was assessed by scanning 14-16 sections of each animal covering the entire transduced striatum using a 20x objective in an Axio Scan.Z1 Slide Scanner microscope. This number was calculated automatically in a blind manner using ImageJ (NIH, USA). Considering the striatal hemisphere volume of 10.75 mm^3^ (ref. [26]) and an average of 20% transduction area per hemisphere, the total number of ataxin-2 transduced cells in the striatum was used to calculate the number of neurons transduced with ataxin-2 per mm^3^.

### Western blot

Cell extracts were lysed in 10x RIPA solution (Merck Millipore) and or mouse striatal punches were homogenized in a urea/DTT solution, both containing a cocktail of protease inhibitors (Roche pharmaceuticals), followed ultrasound sonication. Protein concentration levels were determined using Pierce™ BCA Protein Assay Kit (Thermo Scientific) for cell lysates and using NZYBradford reagent (Nzytech) for mouse samples. Sixty to eighty protein µg were resolved in SDS-polyacrylamide gels (7.5% and 12%). Followed by protein transfer to PVDF membrane (Merck Millipore) and antibody probing using the following antibodies: mouse anti-ataxin-2 (1:1000, ref. 611378, BD Biosciences); mouse anti-IC2 (polyQ chain) (1:1000, ref. MAB1574, Merck Millipore); rabbit anti-LC3B (1:1000, ref. NB100-2220, Novus); rabbit anti-SQSTM1 (1:1000, ref. 5114S, Cell Signaling); mouse anti-β-actin (1:5000, ref. A5316, Sigma Aldrich); rabbit anti-DARP-32 (1:1000, ref. AB10518, Merck Millipore). Optical densiometric analysis was carried out using Image J software (USA).

### Quantitative real-time PCR

Total RNA from mouse striatal punches was extracted with NZY Total RNA Isolation kit (Nzytech), after a first step of Trizol (Invitrogen) tissue dissociation and RNA/DNA/protein chloroform separation. RNA concentration and purity were assessed using the NanoDropTM 2000 (Thermo Scientific). cDNA molecules of 1 µg of RNA were synthesized according to iScript cDNA kit (Bio-Rad) in a 96-well thermocycle (Bio-Rad). For quantitative RT-PCR, it was used SsoAdvanced™ Universal SYBR® Green Supermix (Bio-Rad) in a CFX96 Touch Real-Time PCR Detection System (Bio-Rad). The Mouse Autophagy Primer Library (MATPL-1, Biomol), containing primers for 88 genes related to autophagy and for 8 housekeeping genes, and accordingly RT-PCR conditions were used. mRNA expression levels relative to mRNA gene control were determined using amplification values.

### Statistical analysis

All statistical analysis was performed using Student’s *t*-test or one-way ANOVA followed by post hoc Tukey’s multiple comparison test using GraphPad software (La Jolla, USA). Results are normally expressed as mean ± SEM. Significant thresholds were set at *P* < 0.05, *P* < 0.01, *P* < 0.001 and *P* < 0.0001, as defined in the text.

## Results

### Overexpression of mutant ataxin-2 leads to neuropathological and behavior abnormalities SCA2-related

**Fig. 1:**
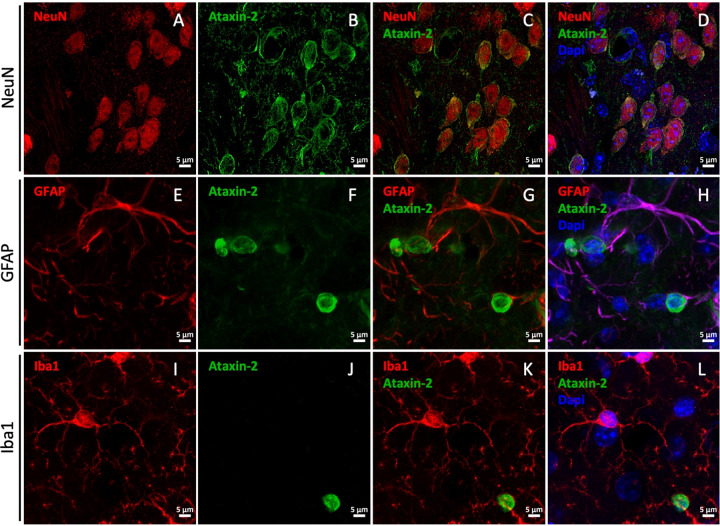
Lentiviral vectors (LVs) encoding for ataxin-2 specifically transduces striatal neurons. LVs encoding for human wild-type ataxin-2 (Atx2WT) and mutant ataxin-2 (Atx2MUT) were injected in mice striatum under the control of neuron-specific PGK promoter. Brain sections were immunoassayed for ataxin-2 protein, the nuclear neuronal marker NeuN, and the glial markers GFAP and Iba1. **A**–**D** Confocal microscopy revealed that ataxin-2 is expressed in NeuN positive neurons, surrounding the nucleus of these cells. On the other hand, GFAP marker and Iba1 co-staining showed that LVs do not transduce glial cells, as ataxin-2 expression does not co-localized with astrocytes (**E**–**H**) or microglia (**I**–**L**).

**Fig. 2:**
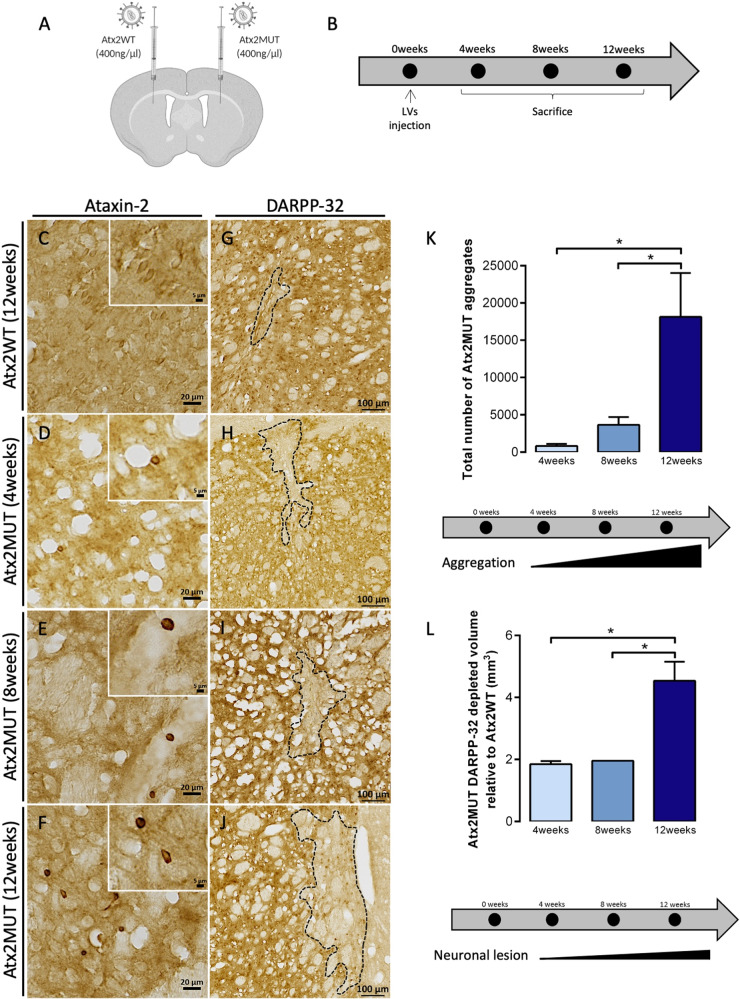
The expression of mutant ataxin-2 in the striatum induces aggregation and loss of neuronal marker. **A** Animals received a stereotaxic injection with lentiviral vectors (LVs) encoding for human wild-type ataxin-2 (Atx2WT) and mutant ataxin-2 (Atx2MUT) in left and right striatum hemisphere, respectively. **B** Animals were divided in three groups according to the time of ataxin-2 expression, for 4-, 8- and 12- weeks after injection. **C** Immunohistochemistry of brain sections revealed a general diffuse signal for ataxin-2 protein expression in Atx2WT hemisphere. In contrast, the overexpression of Atx2MUT resulted in ataxin-2 aggregates at 4 (**D**), 8 (**E**), and 12 (**F**) weeks of experiment. **K** The quantification of the total number of mutant aggregates showed a significant increase over time of ataxin-2 expression, reaching high levels at 12 weeks (values are expressed as mean ± SEM *n* = 3 per timepoint; *P < 0.05; one-way ANOVA followed by post hoc Tukey’s multiple comparisons test). **G** Immunohistochemistry for DARPP-32 neuronal marker showed that Atx2WT results in reduced marker loss, whereas Atx2MUT induces a higher depletion of staining for DARPP-32 either at 4- and 8-weeks post injection (**H**, **I**). These alterations are particularly high at 12 weeks (**J**, **L**) (values are expressed as mean ± SEM *n* = 3 (4-, 8-weeks) and *n* = 8 (12-weeks); *P < 0.05; one-way ANOVA followed by post hoc Tukey’s multiple comparison test).

The development and characterization of adequate non-human models of disease is crucial to elucidate disease mechanisms and to investigate new therapies. For SCA2, different cellular and animal models have been developed [27]. While the use of these models has several advantages, often they only display disease features in advanced ages, which sometimes conditionate the development of research projects in a reasonable timeline. The classical view is that SCA2 selectively impacts the cerebellum, leading to poor movement coordination as the disease progresses. However, clinical evidence from the past 10 years shows that other regions are affected and are the underlying causes for non-ataxia symptoms [6]. Therefore, we aimed to develop a new model based in the expression of mutant ataxin-2 through LVs in the striatum region. A similar approach has been used for Huntington’s disease and MJD (ref. [28, 29]). Previously, it was shown that LVs have a high tropism to transduce neurons [19]. Accordingly, we showed that LVs promote the expression of ataxin-2 in striatal neurons, stained for the neuronal nuclear marker NeuN, regardless of the length of the polyglutamine expansion (Fig. 1A–D). Moreover, LVs did not transduce glial cells, such as astrocytes or microglia, as co-localization between ataxin-2 and the glial markers GFAP and Iba1 was not observed (Fig. 1E–L). Additionally, ataxin-2 expression was under the control of the mouse phosphoglycerate kinase 1 (PGK) promoter, which allows long-term and sustained transduction of striatum neurons [24, 29]. To develop the striatal lentiviral model for SCA2, LVs encoding for human wild-type ataxin-2 (Atx2WT) were injected in mice left striatum, while mutant ataxin-2-encoding vectors (Atx2MUT) were injected in the contralateral hemisphere of the same animal, through stereotaxic surgery (Fig. 2A). These constructs were previously validated in N2A cells, in which we observed the presence of ataxin-2 aggregates upon transfection with Atx2MUT (Supplementary fig. 1). First, we analyzed a group of injected animals to confirm the expression of ataxin-2 in both hemispheres through western blot analysis of striatal punches (Supplementary fig. 2A, B). No differences were found in the expression of Atx2WT and Atx2MUT. For immunohistochemical analysis, the injected animals were divided into three different groups and sacrificed at 4-, 8- and 12-weeks post injection (Fig. 2B). In these animals, we quantified the number of transduced neurons in the striatum per mm^3^, by analyzing the transduced sections and automatically counting the ataxin-2 positive cells. In the same line as what was observed in the western blot analysis, no differences in the expression between Atx2WT (20,609 ± 2091) and Atx2MUT (22,656 ± 1,792) was observed, thus suggesting similar levels of LVs transduction (Supplementary fig. 2C–G). Mutant ataxin-2 protein is prone to aggregate and form cytoplasmic insoluble inclusions that are found in patients’ brain tissue [30]. Immunohistochemistry analysis for ataxin-2 revealed a diffuse staining across Atx2WT injected region (Fig. 2C), while we detected the presence of ataxin-2 aggregates in the Atx2MUT injected hemisphere (Fig. 2D–F). The number of aggregates increased significantly over time, reaching the maximum at 12 weeks post injection (Fig. 2K). We also confirmed, through confocal microscopy, that ataxin-2 aggregates are located in cells cytoplasm surrounding the nucleus, in line to what was reported in previous studies [31] (Supplementary fig. 3). In the MJD lentiviral model, the expression of mutant ataxin-3 resulted in the loss of neuronal markers [29]. In line with this, we found that the expression of Atx2MUT led to a depletion of DARPP-32 marker, being the volume of neuronal loss significantly higher at 12 weeks post injection compared to the 4- and 8- weeks timepoints (Fig. 2H–J, L). In the Atx2WT hemisphere there is a residual DARPP-32 staining depletion (Fig. 2G), although it was lower than the marker loss reported upon an injection with PBS (ref. [32]). Importantly, the levels of DARPP-32 loss were similar across all time points (Supplementary fig. 4). These results show that the expression of mutant ataxin-2, induces neuropathological alterations similar to those observed in SCA2 patients [33, 34].

Neuronal degeneration in different brain regions is typically correlated with behavioral manifestations. Particularly, several studies have shown that striatal dysfunction could lead to a wide range of cognitive and behavioral signs including affective and motivational disturbances, impaired reward processing and anxiety [35]. For that, we injected animals unilaterally with either Atx2WT or Atx2MUT and subjected them to open field test, comparing them also to WT animals. At four weeks post injection, all groups showed similar results in distances covered, zones explored and rearing behavior (Supplementary fig. 5). However, at eight weeks post injection we observed a hyperactive behavior in the Atx2MUT-injected animals compared to the animals injected with Atx2WT or with non-injected animals (Supplementary fig. 6). Specifically, mice expressing expanded ataxin-2 explored longer distances and performed more rearing compared to control groups, which could be suggestive of a hyperactive exploratory behavior [36].

Altogether, these results show that the expression of mutant ataxin-2 in the striatum leads to the development of behavior and neuropathological abnormalities SCA2-related.

### The expression of mutant ataxin-2 leads to neuroinflammation and cell death

**Fig. 3:**
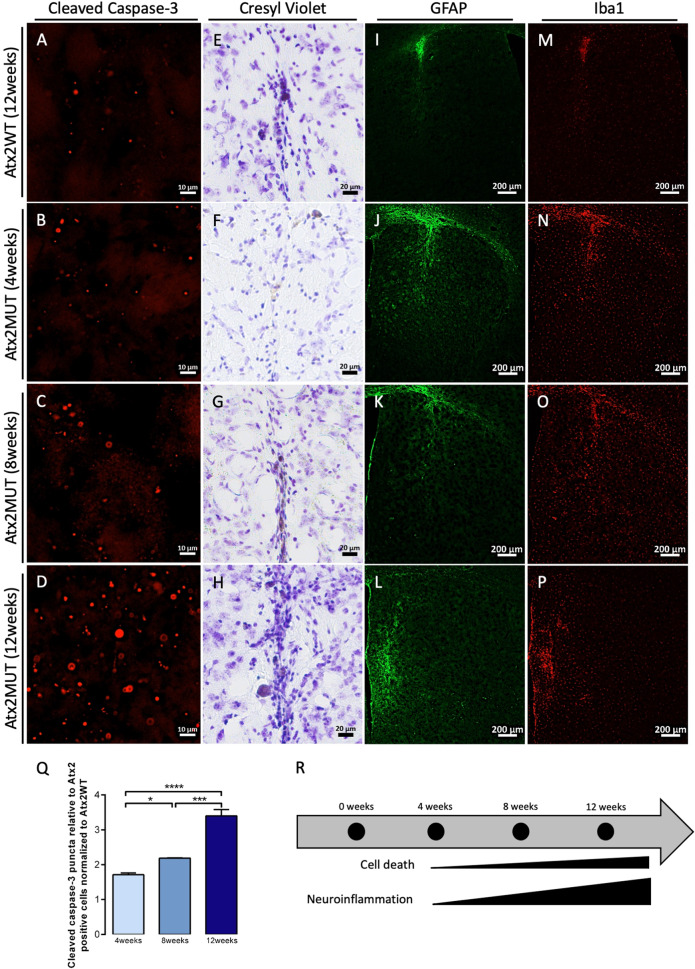
The expression of mutant ataxin-2 leads to cell death and neuroinflammation. Cleaved caspase-3 immunostaining of mouse striatum sections injected with Atx2WT at 12 weeks (**A**) and Atx2MUT at 4-, 8- and 12- weeks postinjection (**B**–**D**). Quantification of cleaved caspase-3 puncta relative to Atx2 positive cells showed that expression of Atx2MUT in the mouse striatum leads to a significant increase of cleaved caspase-3 over the different timepoints, suggesting an increase in apoptosis (**Q**) (values are expressed as mean ± SEM *n* = 3 per timepoint and normalized to Atx2WT; *P < 0.05, ***P < 0.001, ****P < 0.0001; one-way ANOVA followed by post hoc Tukey’s multiple comparison test). **E**–**H** Cresyl violet staining revealed that expression of Atx2MUT resulted in increased presence of pycnotic nuclei structures over time, suggesting cell lesion and striatal degeneration (**R**). Immunohistochemistry for GFAP (astrocytes marker) and Iba-1 (microglia marker) revealed low levels of astrogliosis (**I**) and microgliosis (**M**) upon expression of Atx2WT. In contrast, the overexpression of Atx2MUT induced an inflammatory response, highlighted by increased staining of GFAP (**J**–**L**) and Iba-1 (**N**–**P**) at 4-, 8- and 12-weeks after disease beginning. These neuropathological alterations are higher at 12-weeks of experiment (**R**).

Neuronal death is the ultimate outcome of the molecular events underlying the SCA2 neuropathological process, which might be related to apoptosis. Therefore, in the injected animals we analyzed cleaved caspase-3 labeling (Fig. 3A–D), the active form of caspase-3 protein, which plays a critical role in apoptosis [37]. We observed that the expression of Atx2MUT led to a significant increase in cleaved caspase-3 puncta over the different timepoints of the experiment, suggesting an increase in cell death through apoptosis (Fig. 3Q). Moreover, the formation of pycnotic nuclei structures occurs during the apoptotic process, as chromatin becomes condensed [38]. To assess the presence of these structures upon Atx2MUT expression, we performed a cresyl violet staining of striatal sections. In line with the results depicted above, we observed an increased presence of pycnotic nuclei over time of Atx2MUT expression, suggesting possible cell injury and striatal degeneration (Fig. 3E–H).

Neuroinflammation has been reported in polyQ diseases, as well as in other neurodegenerative disorders [39, 40]. In SCA2, reactive astrocytes and microglia, characterized by the expression of GFAP and Iba1 markers, respectively, were observed in different brain tissues of affected individuals [2, 41, 42]. In line with this, we found that Atx2MUT resulted in marked astrogliosis (Fig. 3J–L) and microgliosis (Fig. 3N–P) across mice striata, compared to a minimal presence of reactive astrocytes and microglia in the needle insertion site in the Atx2WT injected hemisphere (Fig. 3I, M). The triggering of neuroinflammation by Atx2MUT is highlighted by the fact that surgically associated gliosis around the injection site is typically reduced over time, while we found that inflammatory response markers reactivity in Atx2MUT injected hemisphere increased over time (Fig. 3M). Overall, these results indicate that expression of mutant ataxin-2 leads to cell injury and degeneration in mice striatum, while also inducing neuroinflammation.

### Autophagy pathway is impaired in SCA2 models

**Fig. 4:**
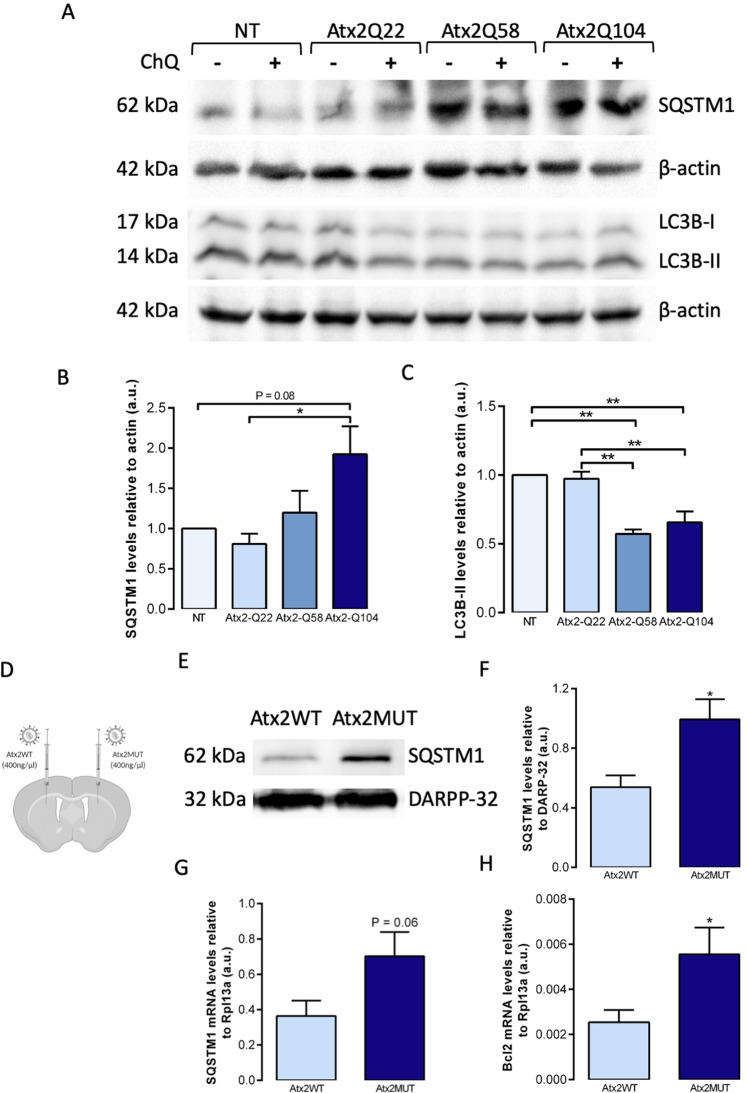
Autophagic markers levels are altered in SCA2 models. Cultured cells from mouse neuroblastoma cell line (Neuro-2A) were transfected either with human wild-type ataxin-2 or mutant ataxin-2 forms, and cells extracts were collected 48h after transfection. **A** Representative western blot analysis of autophagic markers, revealed increased levels of SQSTM1 (**B**) and decreased levels of LC3B-II (**C**) upon expression of mutant ataxin-2, compared to non-transfected (NT) and wild-type ataxin-2 conditions. Data are expressed relative to β-actin levels (*n* = 3 independent experiments). **D** Striatal punches of animals injected with Atx2WT and Atx2MUT were collected for protein and RNA extraction. **E** Western blot analysis showed significantly increased levels of SQSTM1 in the presence of Atx2MUT compared to Atx2WT mice (**F**). Results are expressed relative to DARPP-32 levels (*n* = 8). Quantitative RT-PCR reaction indicated upregulation of SQSTM1 (**G**) and Bcl2 (**H**) mRNA gene levels in Atx2MUT animals compared to control. Results are expressed relative to Rpl13a levels (*n* = 6). Values are expressed as mean ± SEM; *P < 0.05, **P < 0.01; unpaired Student’s *t-*test*;* one-way ANOVA followed by post hoc Tukey’s multiple comparison test).

Several studies have already shown the involvement of autophagy in polyQ pathologies [13], however evident dysfunction of this pathway in SCA2 is still unclear. Thus, we aimed at studying autophagic markers in several SCA2 models. First, we used Neuro-2A cells transfected with different constructs of human ataxin-2, carrying different number of polyQ repeats: Atx2Q22 (WT form), Atx2Q58 and Atx2Q104 (MUT forms) (ref. [20]). We then analyzed the protein levels of SQSTM1, which recognizes ubiquitinated molecules and direct them to autophagosomes [43], and LC3B, responsible for autophagosome maturation [44] (Fig. 4A). We found increased levels of SQSTM1 when cells were expressing mutant ataxin-2 compared to control condition (Fig. 4B). Regarding LC3B protein, we observed a significant decrease in the LC3B-II levels when cells were expressing mutant ataxin-2 forms compared to wild-type ataxin-2 transfected and non-transfected cells (Fig. 4C). These alterations suggest a dysregulation of autophagy pathway upon mutant ataxin-2 expression. Importantly, the levels of these two autophagy markers were similar between non transfected cells and cells expressing the wild-type ataxin-2 protein.

We then investigated the protein levels of SQSTM1 in striatum samples of the lentiviral mouse model (Fig. 4D). In agreement with the in vitro results, we found significantly increased levels of SQSTM1 marker in the hemisphere expressing mutant ataxin-2 compared to control hemisphere (Fig. 4E, F). Additionally, we performed an mRNA array comprising different genes related to the autophagy process. In line with the previous results, we found elevated *SQSTM1* gene expression levels (Fig. 4G), as well as *Bcl2* gene (Fig. 4H) in the hemisphere expressing mutant ataxin-2, compared to the samples expressing the wild-type ataxin-2 protein. Altogether, these data suggest that expression of mutant ataxin-2 leads to abnormal alterations in the autophagy pathway.

### Abnormal accumulation of autophagic markers is detected in SCA2 patients’ brain tissue

**Fig. 5:**
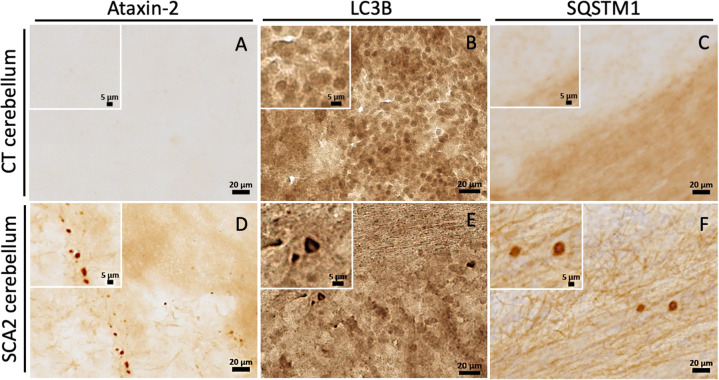
Autophagy markers abnormally accumulate in SCA2 patients’ brain. Immunohistochemical analysis of cerebellum post-mortem brain sections of SCA2 patients and healthy controls. **A** Staining for ataxin-2 showed no immunoreactivity in cerebellum tissue of healthy control. **D** On the other hand, staining for ataxin-2 revealed the presence of aggregated structures in cerebellum of SCA2 brain, indicating a disease state. Staining for LC3B and SQSTM1 autophagic markers demonstrated abnormal puncta accumulation of these proteins in SCA2 cerebellum (**E**, **F**), compared to healthy individual tissues (**B**, **C**).

Several studies have already demonstrated accumulation of autophagic vesicles and markers in neuronal tissues of individuals suffering from polyQ [14]. So far, besides the analysis of SCA2 patients peripheral blood cells [17], no evidences of autophagic dysfunction were described in SCA2 patients’ brain. Upon microscopy analysis, we confirmed the presence of the hallmark mutant ataxin-2 aggregates in the cerebellum of SCA2 patients (Fig.5D), while these aggregates were not observed in healthy individual brain samples (Fig. 5D). Importantly, we detected an abnormal accumulation of LC3B (Fig. 5E) and SQSTM1 (Fig. 5F) markers in SCA2 patients’ brain tissues, comparing to the expression observed in non-disease control neuronal tissue (Fig. 5B, C). Furthermore, this abnormal accumulation of autophagy markers seems also to be observed in striatum tissue from SCA2 patients, compared to healthy individuals (Supplementary fig. 7). These important pathological findings show to our knowledge the first evidence of an autophagic impairment in the brain of SCA2 patients.

### Pharmacologic activation of autophagy through cordycepin mitigates SCA2-associated abnormalities

**Fig. 6:**
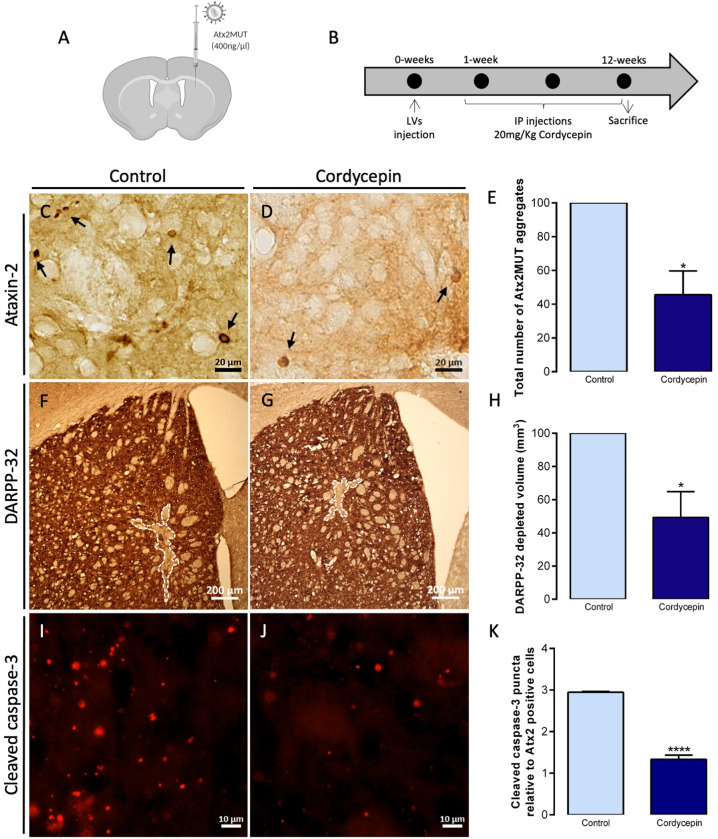
Autophagy upregulation through cordycepin leads to reduced aggregation and neuronal marker loss in the SCA2 striatal mouse model. **A**, **B** Lentiviral mouse model, expressing Atx2MUT in the right striatum hemisphere, was intraperitoneally administered either with the vehicle (NaCl 0.1%) or 20 mg/Kg of cordycepin (in DMSO-NaCl 0.1% solution) for 12 weeks. **C**–**E** Immunohistochemistry of brain sections for ataxin-2 revealed a decrease in the total number of mutant ataxin-2 aggregates upon cordycepin treatment. **F**–**H** Immunohistochemistry staining of DARPP-32 neuronal marker showed a reduction in the depleted volume in treated animals compared to the control group. **I**–**K** Cordycepin treatment resulted in a significant decrease of cleaved caspase-3 puncta relative to ataxin-2 positive cells, compared to non-treated animals (values are expressed as mean ± SEM *n* = 3 (control) and *n* = 4 (cordycepin) relative to control group mean; *P < 0.05, *P < 0.0001; unpaired Student’s *t-*test).

As post-mitotic cells, neurons rely on efficient degradation systems to maintain protein homeostasis [45]. It was shown that dysfunction of these systems, particularly autophagy, contribute to the pathogenesis of neurodegenerative disorders [15]. In line with these studies, we show that the pharmacological inhibition of autophagy results in an increase of the accumulation of aggregates of either wild-type or mutant ataxin-2 (Supplementary fig. 8). Thus, we next aimed to rescue neuropathological abnormalities associated with SCA2, by molecularly inducing autophagy, using the bioactive compound cordycepin or 3′-deoxyadenosine. We have previously shown that cordycepin induces activation of adenosine monophosphate-activated protein kinase (AMPK) pathway, impacting the autophagy process [16]. Moreover, we observed that cordycepin resulted in decreased levels of LC3B-II and SQSTM1, suggesting an increase of autophagic clearance. Importantly, cordycepin treatment led to mitigation of neuropathological and motor deficits observed in mouse models of MJD. In this study, using Neuro-2A cells we found that 20 μM cordycepin treatment led to a significant reduction in the number of cells with mutant ataxin-2 aggregates (Supplementary fig. 9). Next, we went to analyze cordycepin impact in the SCA2 striatal lentiviral mouse model. Briefly, mice expressing Atx2MUT (Fig. 6A) were treated with intraperitoneal injections of 20 mg/Kg of cordycepin for 12 weeks, while control animals received the saline vehicle (Fig. 6B). We found that cordycepin administration resulted in a significant decrease in the number of ataxin-2 aggregates, compared to the control animals (Fig. 6C–E). Moreover, we performed a short-term treatment of four weeks with intraperitoneal injections of 20 mg/Kg of cordycepin or the saline vehicle being administered in mice expressing Atx2MUT to assess ataxin-2 protein levels (Supplementary fig. 10A). In line with the reduction of aggregates, we found a reduction of ataxin-2 protein levels in the cordycepin-treated animals, compared to the control group (Supplementary fig. 10G). Also, we observed a significant preservation of DARPP-32 neuronal marker staining volume in the cordycepin-treated animals (Fig. 6F–H), compared to non-treated group. In the same line, we found that cordycepin treatment resulted in reduced cleaved caspase-3 puncta relative to ataxin-2 positive cells, compared to saline vehicle treated animals (Fig. 6I–K). In addition, we assessed whether cordycepin treatment could impact the neuroinflammation abnormalities of the SCA2 lentiviral mouse model. In fact, we observed lower levels of GFAP and Iba1 immunoreactivity upon treatment with cordycepin, compared to control animals (Supplementary fig. 10C–G). To further demonstrate that cordycepin is inducing the autophagic activity, Neuro-2A cells were transfected with a plasmid expressing LC3B-GFP and -RFP puncta that refers to autophagosomes or autolysosomes, respectively (Supplementary fig. 11). Cells treated with 20 μM cordycepin showed significant higher levels of LC3B-GFP puncta, like starvation condition, indicating increased levels of autophagosomes and suggesting activation of autophagy. Overall, our results support a beneficial role of cordycepin in SCA2 neuropathology, suggesting the upregulation of autophagy as a potential target to treat this pathology.

## Discussion

SCA2 is an incurable and fatal disease prompting devastating physical and psychological consequences for affected individuals and their families. The causative polyQ-expanded ataxin-2 protein is responsible for the cascade of events that precede neuronal death and degeneration of different brain regions [41]. However, the exact mechanisms underlying SCA2 pathogenesis are still controversial and need further elucidation so that a disease-modifying therapy can be developed. Disease models are fundamental tools to study physiological pathways behind human pathologies. Basic models as yeast, *Caenorhabditis elegans* and *Drosophila melanogaster* allowed the study of ataxin-2 biological roles [46, 47]. On the other hand, more complex models, like murine animals, permit a greater and profound functional knowledge, as well as the investigation of therapeutic solutions. The existing SCA2 mouse models have several advantages and have provided valuable insights in disease research [27, 48–53].

While the most affected brain region in SCA2 patients is the cerebellum, and specifically the Purkinje cells [31], it is now clear that several other regions are also profoundly affected in these patients and responsible for the non-ataxia symptoms that SCA2 patients also display [6]. However, there is a lack of studies focusing on the striatum or other regions affected in SCA2, despite the fact that mutant ataxin-2 inclusions and neurodegeneration were observed in the striatum in patients’ brain [31, 33, 34, 54]. In fact, the involvement of this region in the disease could explain the parkinsonism symptoms observed in several SCA2 patients [55]. Therefore, in this study we aimed at developing a mouse model featuring striatal pathology through the expression of mutant ataxin-2 under the control of neuron-specific PGK promoter mediated by lentiviral vectors, which are characterized by neuronal tropism and long-term stable expression [56].

Using this strategy, we detected mutant ataxin-2 aggregates and loss of a neuronal marker, as early as 4 weeks after the injection of LVs encoding mutant ataxin-2. These neuropathological alterations increased significantly over time and reached maximum at 12 weeks post injection. Such early SCA2 signs were only detected in the Q127 transgenic mice, in which perinuclear aggregates were detected at 4 weeks of disease, although no sights of neuronal loss was found [51]. Moreover, the expression of mutant ataxin-2 resulted in increased levels of cleaved caspase-3 puncta and appearance of condensed chromatin pycnotic nuclei, suggesting cellular injury and death. Further characterization of our model strengthened the role of glial cells in SCA2 pathogenesis. Neuroinflammation actively contributes to increased toxicity and neuronal death in many neurodegenerative disorders [40]. This process is mediated by astrocytes and microglia cells, that secrete neurotrophic factors and cytokines [57]. In fact, reactive astrocytes and microglia are present in affected brain regions of SCA2 patients [42, 58]. Accordingly, in our study, we observed astrogliosis and microgliosis in the mutant ataxin-2 expressing hemisphere, which worsened during disease progression. Therefore, our observations also support the inflammatory response as a potential target to treat this condition. Overall, our data suggests that the striatal lentiviral mouse model is a robust and fast disease progression model, reproducing pathological hallmarks present in SCA2 patients.

In polyQ diseases, the causative expanded proteins tend to aggregate and assemble in large aggregates, recruiting other components including RNA-binding proteins, protein chaperones or transcription factors [9, 59], a process that is exacerbated by the failure in protein degradation systems, such as autophagy [60]. Previously, Wardman and colleagues showed that mutant ataxin-2 is eliminated by autophagy rather than by the proteasome system [61]. Taken this in consideration, in our study we evaluated the autophagic pathway status in SCA2. The expression of expanded-polyQ ataxin-2 in neuronal cells and mouse striatum resulted in increased levels of the receptor SQSTM1, while its mRNA levels were upregulated in the SCA2 lentiviral mouse model. These results are in agreement with previous studies, which also demonstrated SQSTM1 abnormal accumulation in SCA2 transgenic mouse models [18, 62]. These results suggest ineffective lysosomal degradation, as SQSTM1 is also recruited to autophagosomes and is normally cleared at the final steps of the process [63]. Moreover, we found that LC3B-II, which is required for autophagosome formation, is decreased in cells expressing mutant ataxin-2, also supporting an autophagy dysregulation. Although LC3B-II levels were found to be upregulated in SCA2 transgenic mouse models, we observed no differences in the SCA2 lentiviral mouse model [18, 62]. As autophagy is a very dynamic process, both decreased or increased LC3B-II levels could suggest autophagic impairment, therefore other autophagic markers should be also taken into consideration [64]. Additionally, the mRNA levels of the anti-apoptotic factor Bcl2 were upregulated in the SCA2 lentiviral mouse model. Bcl2 binds to Beclin-1 protein, inhibiting autophagy initiation [65]. In this study, we also showed evidence for autophagy dysfunction in the brain of SCA2 patients. The impairment of autophagy is strengthened by an abnormal accumulation of autophagic markers in human SCA2 cerebellum and striatum, which agrees with observations in other neurodegenerative diseases. Altogether, these results suggests that mutant ataxin-2 could impact autophagy, thus exacerbating the accumulation of misfolded proteins in SCA2 disorder.

This work reinforces the crucial role of autophagy in SCA2, which could be a potential target for a therapeutic strategy. In fact, a study using an SCA2 cellular model reported that enhancing autophagy resulted in apoptosis decrease [61]. Here, we tested a pharmacological approach to induce the autophagic pathway in SCA2 cellular and mouse models. Previously, we have shown that cordycepin has the ability to improve several neuropathological and behavior deficits in MJD mouse models [65]. In the present study we also observed a neuroprotective effect using a long-term cordycepin treatment in the SCA2 lentiviral mouse model, resulting in decreased mutant ataxin-2 aggregation, preservation of the neuronal loss and reduced cell death.

In conclusion, in this study we developed a new mouse model that bear resemblance to some of the neuropathological hallmarks observed in SCA2 patients, thus constituting a useful model to study disease mechanisms and therapeutic strategies. Importantly, we provide evidence for the dysregulation of the autophagic pathway, in mouse models and SCA2 patients brain samples, and that autophagy upregulation could be a potential therapeutic target for SCA2.

## Supplementary information

Supplementary figures

Emails from authors confirming author list

## Data Availability

The datasets used and/or analyzed during the current study are available from the corresponding author on reasonable request.
