# Supplementary material for: Autophagy in Spinocerebellar ataxia type 2, a dysregulated pathway, and a target for therapy
Source: Cell Death Dis. 2021 Nov 29;12(12):1117. doi: 10.1038/s41419-021-04404-1 (PMC8630050; doi:10.1038/s41419-021-04404-1)
Supplement: Supplementary file 1 — Supplementary figures [file 41419_2021_4404_MOESM1_ESM.docx]

**Supplementary figures**


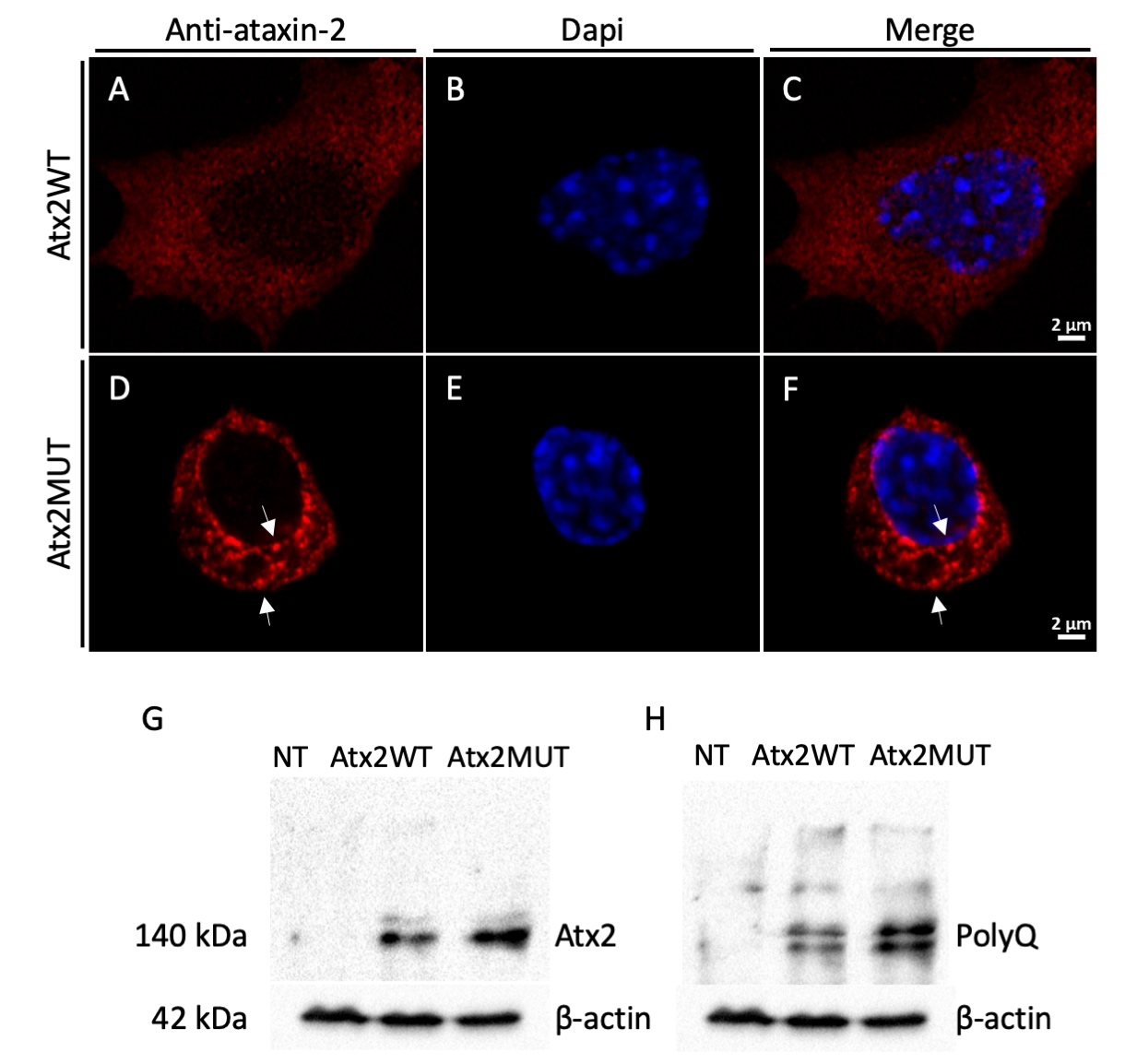


Supplementary fig. 1: **Atx2MUT construct induces aggregation in Neuro-2A cells.** Cultured cells from mouse neuroblastoma cell line (Neuro-2A) were transfected either with human wild-type ataxin-2 (Atx2WT) or mutant ataxin-2 (Atx2MUT). After 48h of transfection, cells were collected and labelled using anti-ataxin-2 and/or anti-1C2 (polyglutamine repeats) antibodies through immunocytochemistry and Western Blot assays, respectively. Confocal microscopy revealed the presence of ataxin-2 positive inclusions in the cytoplasm of cells expressing Atx2MUT (D, E, F) while cells transfected with Atx2WT did not present aggregated structures (A, B, C). Western blot results showed higher levels of polyglutamine chain in Atx2MUT condition compared to Atx2WT or non-transfected cells (NT) (G, H).


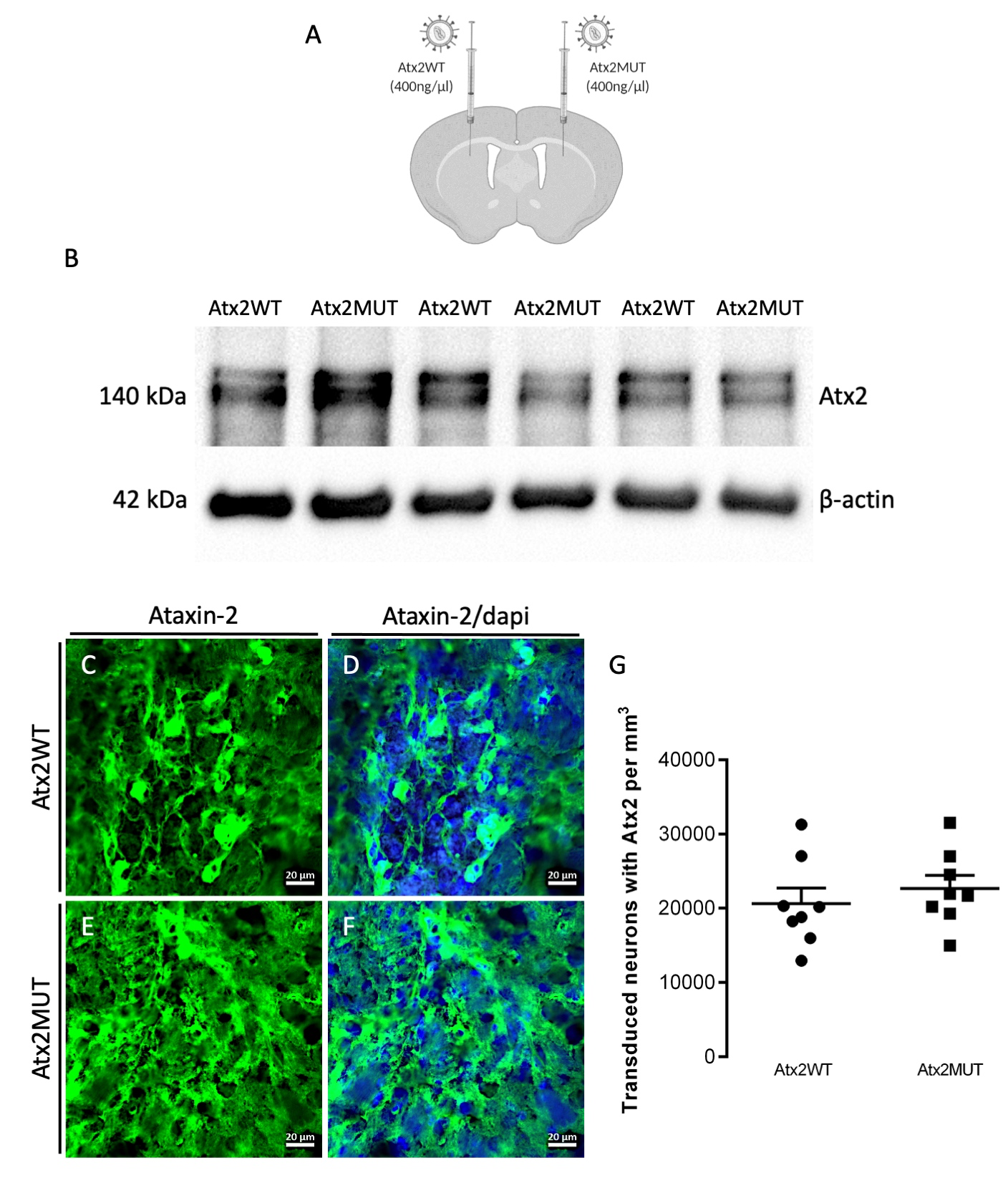


Supplementary fig. 2: **Lentiviral vectors (LVs) encoding for Atx2WT and Atx2MUT leads to similar expression levels of ataxin-2 in the striatum.** (A) Mice were stereotaxically injected with LVs encoding for human wild-type ataxin-2 (Atx2WT) and mutant ataxin-2 (Atx2MUT) under the control of PGK promoter in left and right striatum hemisphere, respectively. Striatal punches were collected 12 weeks after LVs expression and analyzed through Western blot for ataxin-2 protein. (B) We confirmed the expression of ataxin-2 upon LVs administration in both Atx2WT and Atx2MUT hemispheres. (C-F) Histological staining against ataxin-2 of striatum sections (14-16) were scanned in a fluorescence microscope. (G) Upon quantification of the number of striatal neurons transduced with ataxin-2 per mm^3^, no differences were found in the different constructs suggesting equal levels of transduction. Values are expressed as mean ± SEM *n*=8; *unpaired Student’s t-test*.


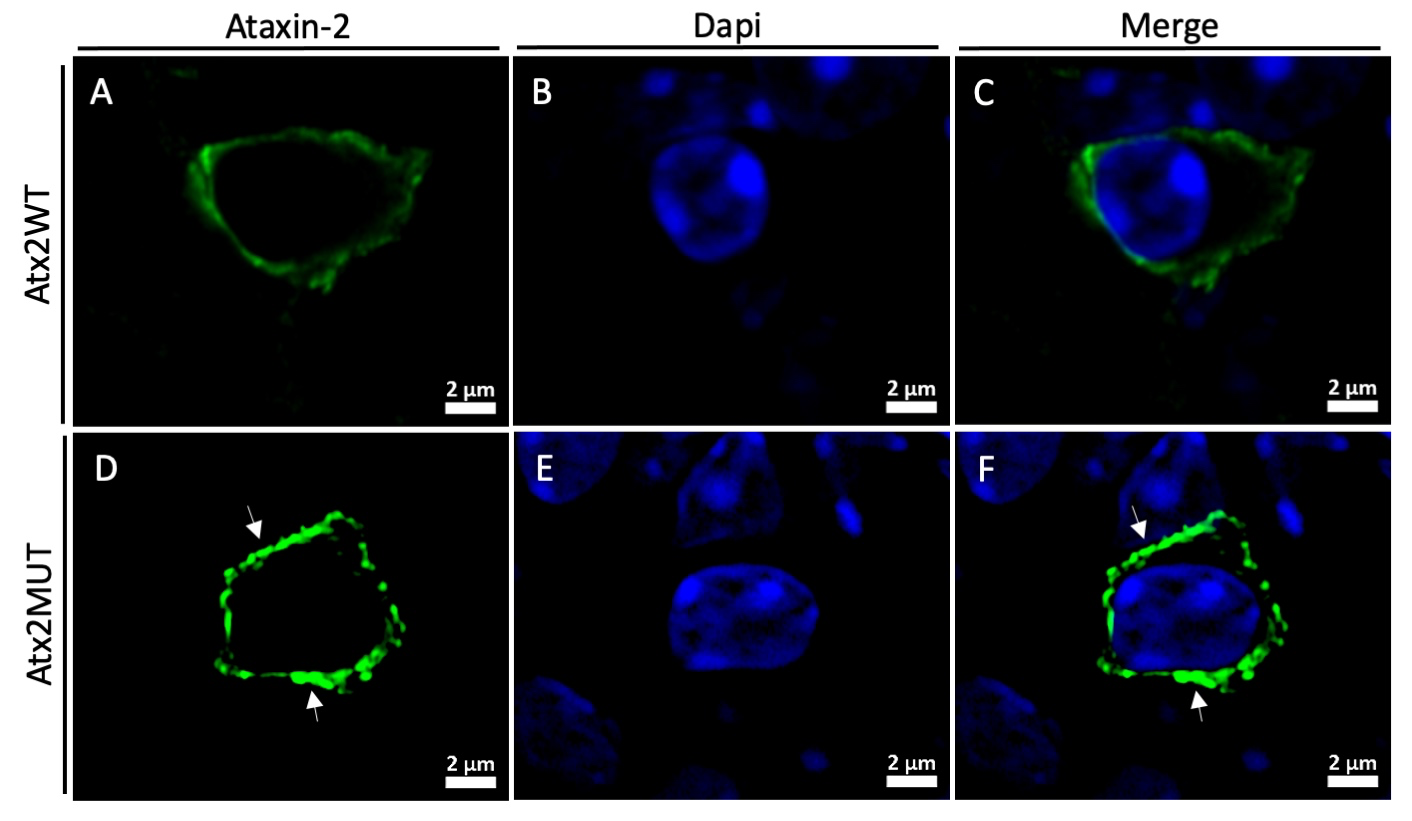


Supplementary fig. 3: **Atx2WT is diffusely expressed in the cytoplasm, while** **Atx2MUT forms cytoplasmic aggregates surrounding the nucleus.** Confocal microscopy revealed that Atx2WT is localized along cells cytoplasm (A, B, C), while the presence of Atx2MUT results in aggregated structures (indicated by arrows) inside cells cytoplasm in mice striatum (D, E, F), upon injection of lentiviral vectors encoding for wild-type or mutant ataxin-2 and 12 weeks after injection.


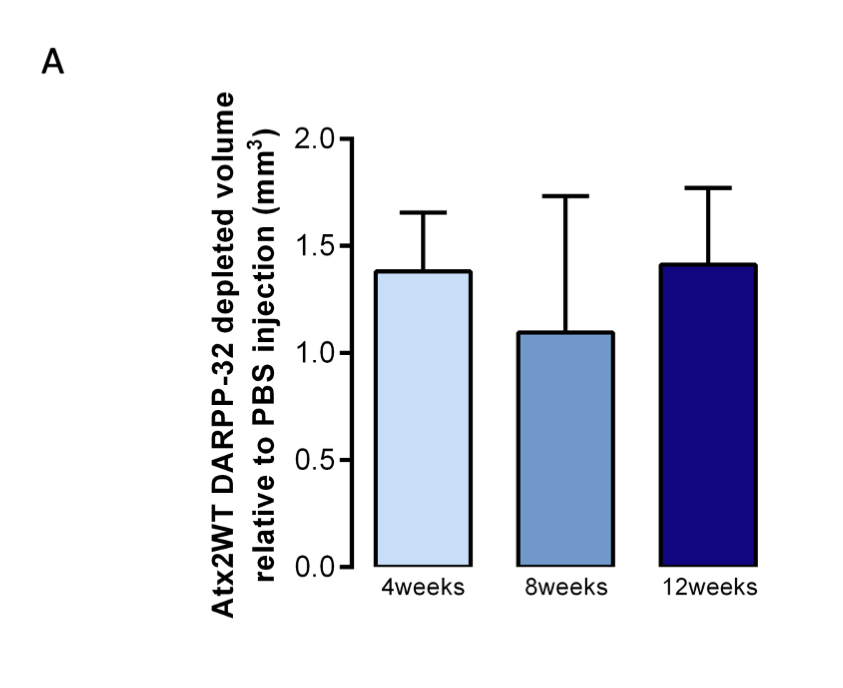


Supplementary fig. 4: **The expression of Atx2-WT mediated by LVs in the striatum leads to a similar volume of DARPP-32 loss at 4-, 8- and 12-weeks.** (A) Quantification of DARPP-32 neuronal marker depleted volume in mice left hemisphere injected with LVs encoding for human wild-type ataxin-2 (Atx2WT). We observed similar levels of DARPP-32 depleted staining at 4-, 8- and 12-weeks after injection, indicating that expression of Atx2WT does not aggravate neuronal marker loss over time (values are expressed as mean ± SEM relative to PBS injection *n*=3; one-way ANOVA followed by *post hoc* Tukey's multiple comparisons test).


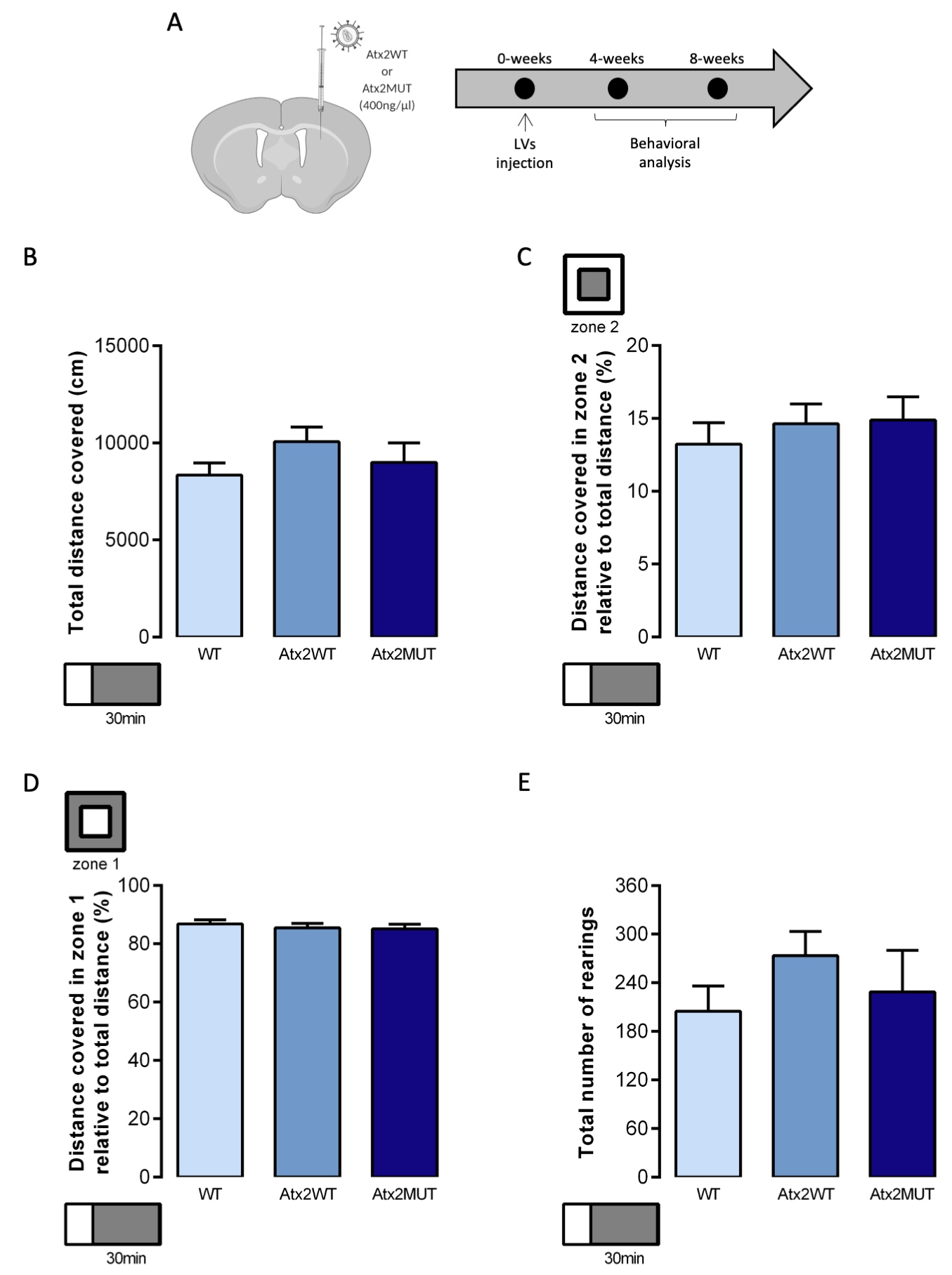


Supplementary fig. 5: **The expression of Atx2MUT mediated by LVs in the mouse striatum does not induce behavioral alterations at 4 weeks post injection**. Wild-type animals (*n*=5) or animals injected with Atx2WT (*n*=9) or Atx2MUT (*n*=8) were subjected to the open field test for 40min (10min of adaptation/30min of test analysis) at 4-weeks post-injection. Mice expressing Atx2MUT covered the same distance in the open field box compared to Atx2WT and WT groups (A). Moreover, no differences were observed between the three groups of animals regarding the distances covered in central zone (zone 2) (C) or the periphery (zone 1) (D). The analysis of the number of rearings during the test indicated that animals expressing mutant ataxin-2 showed similar rearing activity compared to the other two groups. The results suggest no alterations in behavioral activity at 4-weeks upon mutant ataxin-2 expression. Values are expressed as mean ± SEM; one-way ANOVA followed by *post hoc* Tukey's multiple comparisons test.


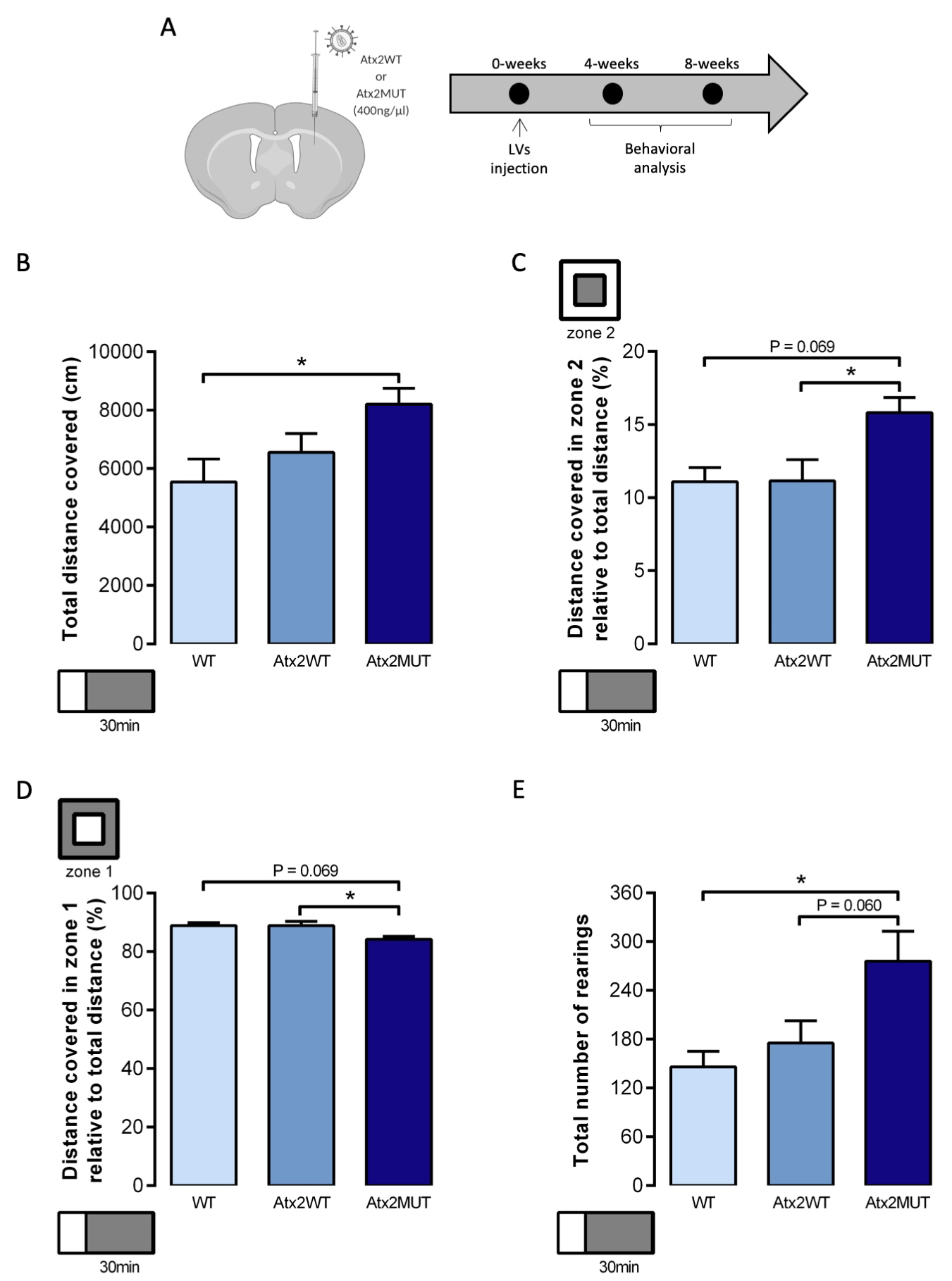


Supplementary fig. 6: **The** **expression of Atx2MUT in the mouse striatum leads to hyperactive behavioral alterations 8 weeks after injection**. Wild-type animals (*n*=5) or animals injected with Atx2WT (*n*=9) or Atx2MUT (*n*=8) were subjected to the open field test for 40min (10min of adaptation/30min of test analysis) at 8-weeks post-injection. Mice overexpressing Atx2MUT covered a longer total distance compared to Atx2WT and WT groups (A). The animals injected with the mutant form of the protein preferred the central area of the arena (zone 2) (B) instead of the periphery zone 1) (C), as indicated by the distance traveled in each zone relative to the total distance. The analysis of the rearing behavior during the test indicated that animals expressing mutant ataxin-2 did a higher number of rearings compared to the other two groups. Values are expressed as mean ± SEM; **P* ˂ 0.05; one-way ANOVA followed by *post hoc* Tukey's multiple comparisons test.


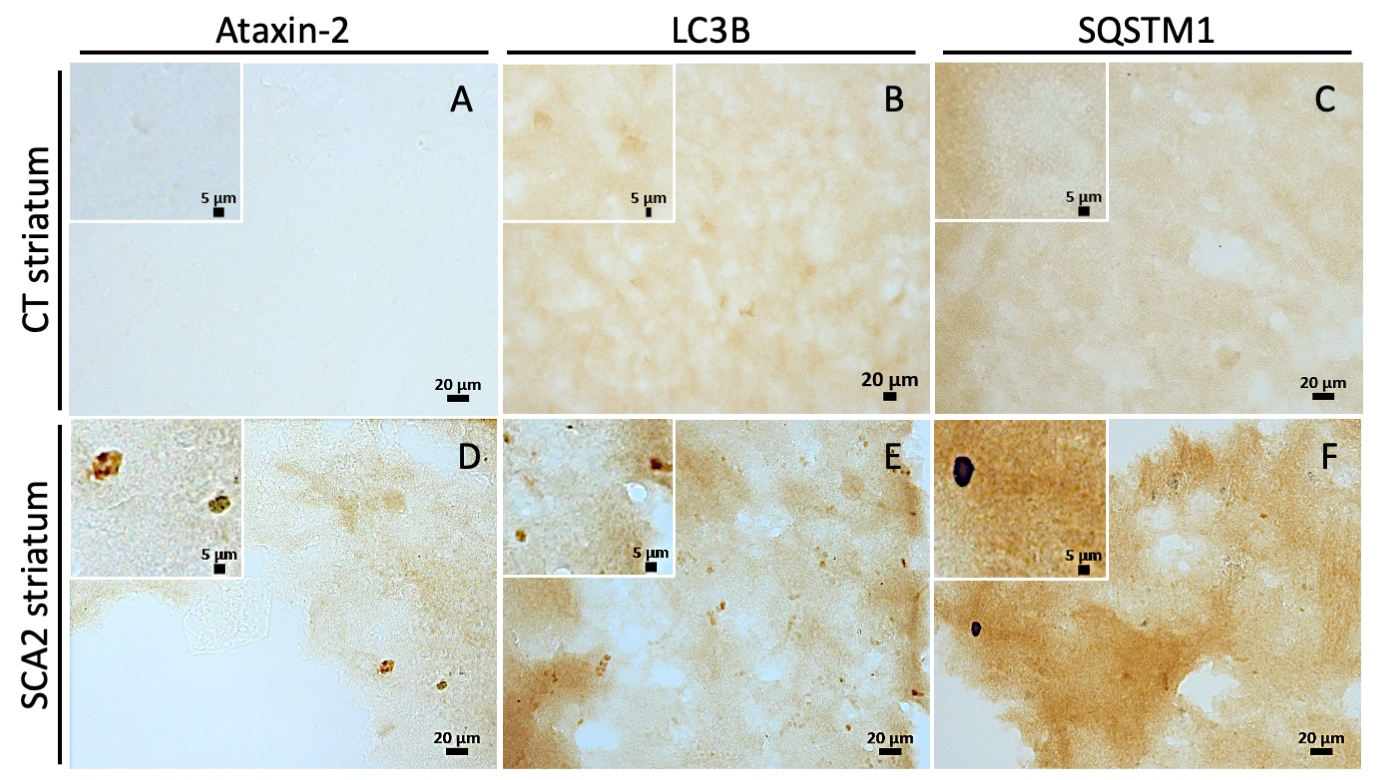


Supplementary fig. 7: **LC3B and SQSTM1 autophagic markers accumulate abnormally in SCA2 patients’ striatum tissue.** Immunohistochemical analysis of striatum *post-mortem* brain samples of SCA2 patients and healthy controls. (A) Staining for ataxin-2 showed no immunoreactivity in striatum of healthy control. (D) On the other hand, staining for ataxin-2 revealed the presence of aggregate-like structures in the striatum, suggesting neuropathological alterations. Staining for LC3B and SQSTM1 autophagic markers shows abnormal puncta accumulation of these proteins in SCA2 striatum (E-F), compared to healthy individual tissues (B-C).


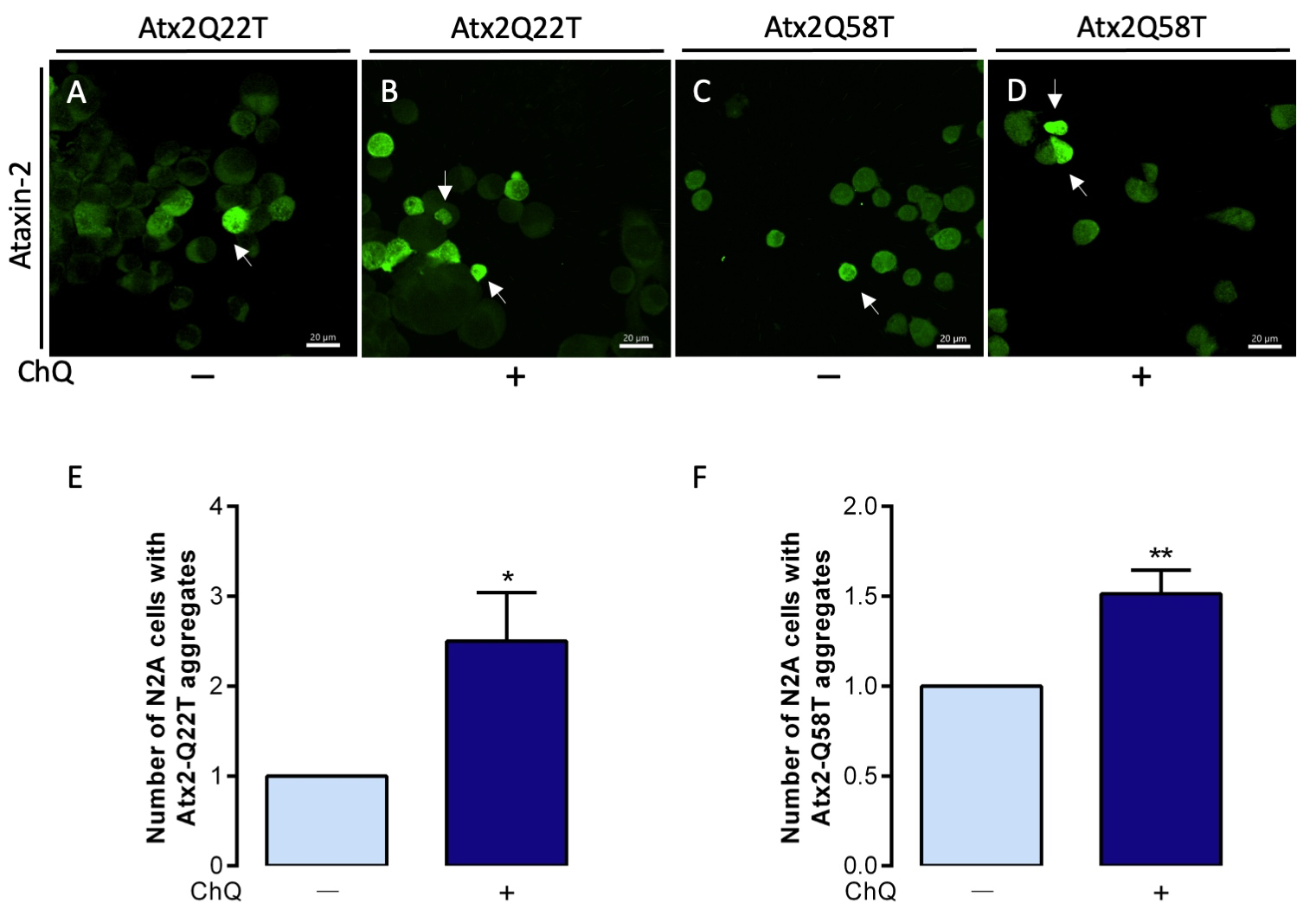


Supplementary fig. 8: **Blocking autophagy promotes ataxin-2 protein aggregation in Neuro-2A cells.** Cultured cells from mouse neuroblastoma cell line (Neuro-2A) were transfected with ataxin-2 truncated form with an EGFP tag carrying normal polyQ segment (22Q – Atx2-Q22T) or mutant polyQ segment (58Q – Atx2-Q58T) for 48h. Chloroquine (ChQ) was added to cells 6h before harvest for analysis. Fluorescence microscopy revealed an increase in the number of cells with either Atx2-Q22T (A, B, E) or Atx2-Q58T (C, D, F) after ChQ treatment. Values are expressed as mean ± SEM *n*=4; **P* ˂ 0.05, ***P* ˂ 0.01; *unpaired Student’s t-test*.


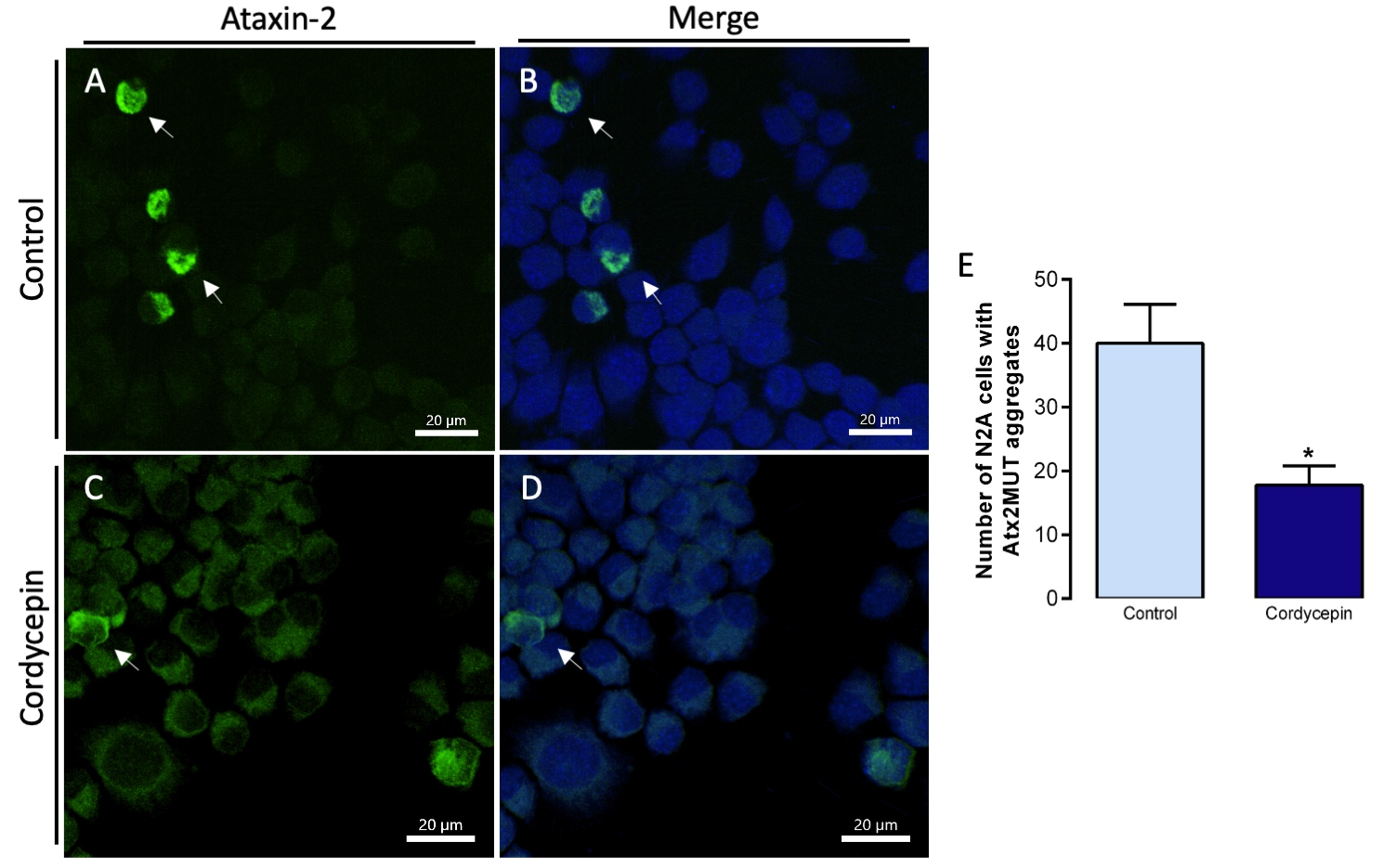


Supplementary fig. 9: **Induction of** **autophagy using cordycepin results in a decrease in the number of Neuro-2A cells with Atx2MUT aggregates.** Cultured cells from mouse neuroblastoma cell line (Neuro-2A) were transfected with Atx2MUT and after 24h received a 20μᴍ cordycepin treatment for 48h. (A-E) Fluorescence microscopy revealed a reduction in the number of cells with mutant ataxin-2 aggregates after cordycepin treatment (*n*=5), compared to control condition (*n*=3) (arrows indicate aggregates; values are expressed as mean ± SEM *P ˂ 0.05; *unpaired Student’s t-test*).


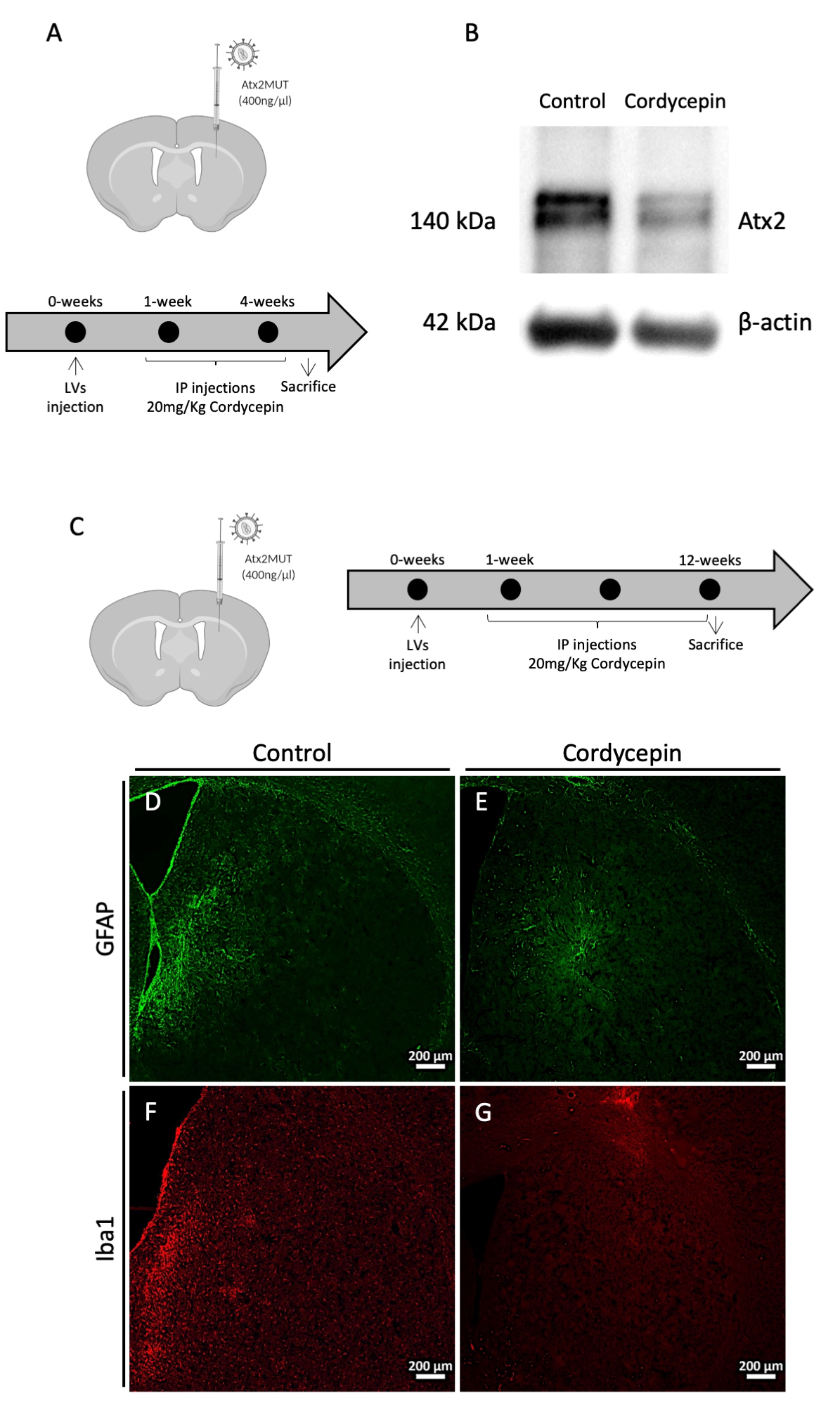


Supplementary fig. 10: **Autophagy activation through cordycepin reduces the levels of mutant ataxin-2 and decreases neuroinflammation** (A) Animlas expressing Atx2MUT in the right striatum hemisphere, were intraperitoneally administered either with the vehicle (NaCl 0.1%, *n*=2) or 20mg/Kg of cordycepin (in DMSO-NaCl 0.1% solution, *n*=2) for 4 weeks. (B) Western blot of striatal punches suggests a reduction of ataxin-2 protein levels upon cordycepin administration. (C) Animals expressing Atx2MUT in the right striatum hemisphere, were intraperitoneally administered either with the vehicle (NaCl 0.1%, *n*=3) or 20mg/Kg of cordycepin (in DMSO-NaCl 0.1% solution, *n*=4) for 12 weeks. (D-G) Immunohistochemistry of brain sections revealed decreased immunoreactivity for GFAP and Iba1 markers upon cordycepin treatment, suggesting reduced levels of neuroinflammation in the treated animals.


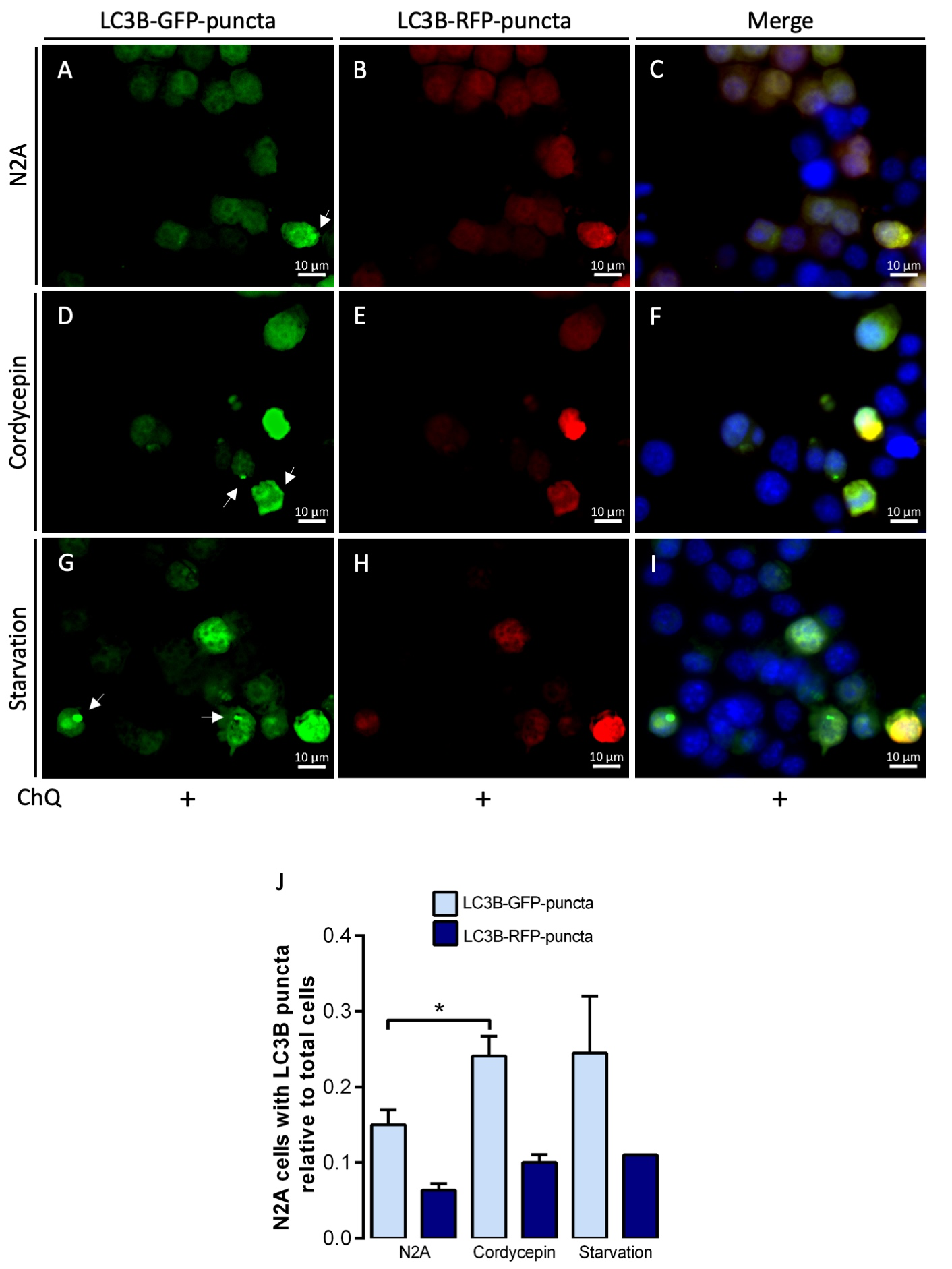


Supplementary fig. 11: **Cordycepin increases LC3B-GFP puncta in Neuro-2A cells.** Cultured cells from mouse neuroblastoma cell line (Neuro-2A) were transfected for 48h with a plasmid expressing the autophagic marker LC3B fused with RFP and GFP tag (ptfLC3-RFP-GFP). Additionally, one condition received a 20µM cordycepin treatment 24h after transfection and a condition of starvation, using Hanks’ Balanced Salt Solution supplemented with 1.8g/L of glucose, was induced 6h prior to cells harvest. Chloroquine (ChQ) was added to all conditions 6h before harvest to block the autophagic process. (A-I) Direct fluorescent microscopy revealed Neuro-2A cells with LC3B-GPF puncta, which represents autophagosomes, as well as LC3B-RFP puncta, which refers to autolysosomes. (J) For each condition, cells with LC3B-GFP or LC3B-RFP puncta were randomly counted in a total of 100-120 transfected cells. Cordycepin treatment (*n*=3) resulted in significantly increased levels of LC3B-GFP puncta relative to non-treated cells (*n*=3), suggesting increased number of autophagosomes and increased autophagic activity. Moreover, these levels were like those found in the starvation condition (*n*=2), a positive control of autophagy activation. Values are expressed as mean ± SEM relative to total number of cells counted; **P* ˂ 0.05; *unpaired Student’s t-test*.
