# Supplementary material for: Autophagy in Spinocerebellar ataxia type 2, a dysregulated pathway, and a target for therapy
Source: Cell Death Dis. 2021 Nov 29;12(12):1117. doi: 10.1038/s41419-021-04404-1 (PMC8630050; doi:10.1038/s41419-021-04404-1)
Supplement: Supplementary file 2 — Emails from authors confirming author list [file 41419_2021_4404_MOESM2_ESM.pdf]

Gmail

URGENTE - Paper Cell death and Disease - CDDIS-21-2430

Adriana do Vale Marcelo <adrianamarcelo11@hotmail.com> 12 de novembro de 2021 às 11:56
Para: Clevio Nobrega <cdnobrega@ualg.pt>, Inês Torquato Afonso <inesta.28@gmail.com>, Ricardo Reis <reisricardo12@hotmail.com>, David Brito <david.v.c.brito@gmail.com>, Rafael Costa <rafael.gm.costa@gmail.com>, Benedita Ferreira <ditaferre@gmail.com>, João Cruzeiro <jc.joaocruzeiro@gmail.com>, "cshenriques@cnc.uc.pt" <cshenriques@cnc.uc.pt>, Carlos Adriano Albuquerque Andrade de Matos <camatos@ualg.pt>, Luis Pereira de Almeida <lpereiradealmeida@gmail.com>, Rui Nobre <rui.jorge.nobre@gmail.com>, ANA RITA TIAGO ROSA <a52713@ualg.pt>, "Ana (Solo) Rosa" <ana.rosa.9603@gmail.com>

Dear all,

I agree with all the alterations.

Best regards,

Adriana Marcelo
PhD Student
PhD in Biomedical Sciences
Department of Biomedical Sciences and Medicine
University of Algarve
Campus de Gambelas
8005-139 Faro, Portugal
Phone: +351289800100, Ext. (2)203301

De: Clevio Nobrega <cdnobrega@ualg.pt>
Enviado: 12 de novembro de 2021 11:39
Para: Adriana do Vale <adrianamarcelo11@hotmail.com>; Inês Torquato Afonso <inesta.28@gmail.com>; Ricardo Reis <reisricardo12@hotmail.com>; David Brito <david.v.c.brito@gmail.com>; Rafael Costa <rafael.gm.costa@gmail.com>; Benedita Ferreira <ditaferre@gmail.com>; João Cruzeiro <jc.joaocruzeiro@gmail.com>; cshenriques@cnc.uc.pt <cshenriques@cnc.uc.pt>; Carlos Adriano Albuquerque Andrade de Matos <camatos@ualg.pt>; Luis Pereira de Almeida <lpereiradealmeida@gmail.com>; Rui Nobre <rui.jorge.nobre@gmail.com>; ANA RITA TIAGO ROSA <a52713@ualg.pt>; Ana (Solo) Rosa <ana.rosa.9603@gmail.com>
Assunto: URGENTE - Paper Cell death and Disease - CDDIS-21-2430

Dear all,
As you know the paper "Autophagy in Spinocerebellar ataxia type 2, a dysregulated pathway, and a target for therapy" by Adriana was accepted in Cell death and disease. As several new experiments were made in this last round of reviews, we include two additional authors (Rafael and David) and changed the order list to include them and reflect the new work made. Now I need that you all confirm that you agree with this alteration.

So please reply by today to this email confirming that you agree with the changes! without this, we cannot proceed with the publication procedure.

Thank you in advance.

Best

CLÉVIO DAVID RODRIGUES **NÓBREGA**

Original authors: Adriana Marcelo,[1,2,3,4] Ana Rosa,[1] João Alves-Cruzeiro,[3] Benedita Ferreira,[1,4] Ricardo Reis,[1,4] Inês Afonso,[1,4] Carina Henriques,[3] Rui J. Nobre,[3] Carlos A. Matos,[1,4] Luís Pereira de Almeida,[3,5] Clévio Nóbrega [1,4,6*]

Final list:Adriana Marcelo,[1,2,3,4], Inês T. Afonso,[1] Ricardo Afonso-Reis,[1,4] David V.C. Brito,[1] Rafael G. Costa,[1,4] Ana Rosa,[1] João Alves-Cruzeiro,[3] Benedita Ferreira,[1,4] Carina Henriques,[3] Rui J. Nobre,[3] Carlos A. Matos,[1,4] Luís Pereira de Almeida,[3,5] Clévio Nóbrega [1,4,6*]

Gmail

Clevio Nobrega <kl3vio@gmail.com>

URGENTE - Paper Cell death and Disease - CDDIS-21-2430

Ana Rosa <ana.rosa.9603@gmail.com> 12 de novembro de 2021 às 13:52
Para: Clevio Nobrega <cdnobrega@ualg.pt>
Cc: Adriana do Vale <adrianamarcelo11@hotmail.com>, Inês Torquato Afonso <inesta.28@gmail.com>, Ricardo Reis <reisricardo12@hotmail.com>, David Brito <david.v.c.brito@gmail.com>, Rafael Costa <rafael.gm.costa@gmail.com>, Benedita Ferreira <ditaferre@gmail.com>, João Cruzeiro <jc.joaocruzeiro@gmail.com>, cshenriques@cnc.uc.pt, Carlos Adriano Albuquerque Andrade de Matos <camatos@ualg.pt>, Luis Pereira de Almeida <lpereiradealmeida@gmail.com>, Rui Nobre <rui.jorge.nobre@gmail.com>, ANA RITA TIAGO ROSA <a52713@ualg.pt>

Dear all,

I agree with the changes in the list of authors.
Best regards,

Ana Rita Rosa

A sexta, 12/11/2021, 11:39, Clevio Nobrega <cdnobrega@ualg.pt> escreveu:

> Dear all,
> As you know the paper "Autophagy in Spinocerebellar ataxia type 2, a dysregulated pathway, and a target for therapy" by Adriana was accepted in Cell death and disease. As several new experiments were made in this last round of reviews, we include two additional authors (Rafael and David) and changed the order list to include them and reflect the new work made. Now I need that you all confirm that you agree with this alteration.
>
> **So please reply by today to this email confirming that you agree with the changes! without this**, we cannot proceed with the publication procedure.
>
> Thank you in advance.
>
> Best
>
> **CLÉVIO** DAVID RODRIGUES **NÓBREGA**
>
> Original authors: Adriana Marcelo,[1,2,3,4] Ana Rosa,[1] João Alves-Cruzeiro,[3] Benedita Ferreira,[1,4] Ricardo Reis,[1,4] Inês Afonso,[1,4] Carina Henriques,[3] Rui J. Nobre,[3] Carlos A. Matos,[1,4] Luís Pereira de Almeida,[3,5] Clévio Nóbrega [1,4,6*]
>
> Final list:Adriana Marcelo,[1,2,3,4], Inês T. Afonso,[1] Ricardo Afonso-Reis,[1,4] David V.C. Brito,[1] Rafael G. Costa,[1,4] Ana Rosa,[1] João Alves-Cruzeiro,[3] Benedita Ferreira,[1,4] Carina Henriques,[3] Rui J. Nobre,[3] Carlos A. Matos,[1,4] Luís Pereira de Almeida,[3,5] Clévio Nóbrega [1,4,6*]

Gmail

Clevio Nobrega <kl3vio@gmail.com>

URGENTE - Paper Cell death and Disease - CDDIS-21-2430

Carina Santos Henriques <cshenriques@cnc.uc.pt> 12 de novembro de 2021 às 12:32
Para: Carlos Matos <camatos@ualg.pt>
Cc: David Brito <david.v.c.brito@gmail.com>, ANA RITA TIAGO ROSA <a52713@ualg.pt>, Adriana do Vale <adrianamarcelo11@hotmail.com>, "Ana (Solo) Rosa" <ana.rosa.9603@gmail.com>, Benedita Ferreira <ditaferre@gmail.com>, Clevio Nobrega <cdnobrega@ualg.pt>, Inês Torquato Afonso <inesta.28@gmail.com>, João Cruzeiro <jc.joaocruzeiro@gmail.com>, Luis Pereira de Almeida <lpereiradealmeida@gmail.com>, Rafael Costa <rafael.gm.costa@gmail.com>, Ricardo Reis <reisricardo12@hotmail.com>, Rui Nobre <rui.jorge.nobre@gmail.com>

Dear all,

I do agree with the alterations regarding the new order of the authors' list.

Best regards,
Carina Henriques

A sexta, 12/11/2021, 12:24, Carlos Matos <camatos@ualg.pt> escreveu:

> I am in agreement with the changes.
>
> All the best,
>
> Carlos Matos
>
> David Brito <david.v.c.brito@gmail.com> escreveu em sex., 12/11/2021 às 12:18 :
>
> > Dear all,
> >
> > I agree with the above-mentioned changes.
> >
> > Best wishes,
> >
> > **David**
> >
> > On Fri, 12 Nov 2021 at 11:39, Clevio Nobrega <cdnobrega@ualg.pt> wrote:
> >
> > > Dear all,
> > > As you know the paper "Autophagy in Spinocerebellar ataxia type 2, a dysregulated pathway, and a target for therapy" by Adriana was accepted in Cell death and disease. As several new experiments were made in this last round of reviews, we include two additional authors (Rafael and David) and changed the order list to include them and reflect the new work made. Now I need that you all confirm that you agree with this alteration.
> > >
> > > **So please reply by today to this email confirming that you agree with the changes! without this**, we cannot proceed with the publication procedure.
> > >
> > > Thank you in advance.
> > >
> > > Best
> > >
> > > **CLÉVIO** DAVID RODRIGUES **NÓBREGA**
> > >
> > > Original authors: Adriana Marcelo,[1,2,3,4] Ana Rosa,[1] João Alves-Cruzeiro,[3] Benedita Ferreira,[1,4] Ricardo Reis,[1,4] Inês Afonso,[1,4] Carina Henriques,[3] Rui J. Nobre,[3] Carlos A. Matos,[1,4] Luís Pereira de Almeida,[3,5] Clévio Nóbrega [1,4,6*]
> > >
> > > Final list:Adriana Marcelo,[1,2,3,4], Inês T. Afonso,[1] Ricardo Afonso-Reis,[1,4] David V.C. Brito,[1] Rafael G. Costa,[1,4] Ana Rosa,[1] João Alves-Cruzeiro,[3] Benedita Ferreira,[1,4] Carina Henriques,[3] Rui J. Nobre,[3] Carlos A. Matos,[1,4] Luís Pereira de Almeida,[3,5] Clévio Nóbrega [1,4,6*]

--
Carlos A. Matos, PhD

Post-doctoral Associate Researcher
Algarve Biomedical Center - Research Institute (ABC-RI)
Faculty of Medicine and Biomedical Sciences
University of Algarve
Faro, Portugal

MNGT Molecular Neuroscience and Gene Therapy Lab
ABC Ri ALGARVE BIOMEDICAL CENTER RESEARCH INSTITUTE
UAlg FMCB UNIVERSIDADE DO ALGARVE FACULDADE DE MEDICINA E CIÊNCIAS BIOMÉDICAS
UAlg UNIVERSIDADE DO ALGARVE

Gmail

Clevio Nobrega <kl3vio@gmail.com>

URGENTE - Paper Cell death and Disease - CDDIS-21-2430

Carlos Matos <camatos@ualg.pt> 12 de novembro de 2021 às 12:24
Para: David Brito <david.v.c.brito@gmail.com>
Cc: ANA RITA TIAGO ROSA <a52713@ualg.pt>, Adriana do Vale <adrianamarcelo11@hotmail.com>, "Ana (Solo) Rosa" <ana.rosa.9603@gmail.com>, Benedita Ferreira <ditaferre@gmail.com>, Clevio Nobrega <cdnobrega@ualg.pt>, Inês Torquato Afonso <inesta.28@gmail.com>, João Cruzeiro <jc.joaocruzeiro@gmail.com>, Luis Pereira de Almeida <lpereiradealmeida@gmail.com>, Rafael Costa <rafael.gm.costa@gmail.com>, Ricardo Reis <reisricardo12@hotmail.com>, Rui Nobre <rui.jorge.nobre@gmail.com>, cshenriques@cnc.uc.pt

I am in agreement with the changes.

All the best,

Carlos Matos

David Brito <david.v.c.brito@gmail.com> escreveu em sex., 12/11/2021 às 12:18 :

> Dear all,
>
> I agree with the above-mentioned changes.
>
> Best wishes,
>
> **David**
>
> On Fri, 12 Nov 2021 at 11:39, Clevio Nobrega <cdnobrega@ualg.pt> wrote:
>
>> Dear all,
>> As you know the paper "Autophagy in Spinocerebellar ataxia type 2, a dysregulated pathway, and a target for therapy" by Adriana was accepted in Cell death and disease. As several new experiments were made in this last round of reviews, we include two additional authors (Rafael and David) and changed the order list to include them and reflect the new work made. Now I need that you all confirm that you agree with this alteration.
>>
>> **So please reply by today to this email confirming that you agree with the changes! without this**, we cannot proceed with the publication procedure.
>>
>> Thank you in advance.
>>
>> Best
>>
>> **CLÉVIO** DAVID RODRIGUES **NÓBREGA**
>>
>> Original authors: Adriana Marcelo,[1,2,3,4] Ana Rosa,[1] João Alves-Cruzeiro,[3] Benedita Ferreira,[1,4] Ricardo Reis,[1,4] Inês Afonso,[1,4] Carina Henriques,[3] Rui J. Nobre,[3] Carlos A. Matos,[1,4] Luís Pereira de Almeida,[3,5] Clévio Nóbrega [1,4,6*]
>>
>> Final list:Adriana Marcelo,[1,2,3,4], Inês T. Afonso,[1] Ricardo Afonso-Reis,[1,4] David V.C. Brito,[1] Rafael G. Costa,[1,4] Ana Rosa,[1] João Alves-Cruzeiro,[3] Benedita Ferreira,[1,4] Carina Henriques,[3] Rui J. Nobre,[3] Carlos A. Matos,[1,4] Luís Pereira de Almeida,[3,5] Clévio Nóbrega [1,4,6*]

--
***Carlos A. Matos**, PhD*

Post-doctoral Associate Researcher
Algarve Biomedical Center - Research Institute (ABC-RI)

Faculty of Medicine and Biomedical Sciences
University of Algarve
Faro, Portugal

Gmail **Clevio Nobrega <kl3vio@gmail.com>**

URGENTE - Paper Cell death and Disease - CDDIS-21-2430

João Cruzeiro <jc.joaocruzeiro@gmail.com> 12 de novembro de 2021 às 13:09
Para: Carina Santos Henriques <cshenriques@cnc.uc.pt>
Cc: Carlos Matos <camatos@ualg.pt>, David Brito <david.v.c.brito@gmail.com>, ANA RITA TIAGO ROSA <a52713@ualg.pt>, Adriana do Vale <adrianamarcelo11@hotmail.com>, "Ana (Solo) Rosa" <ana.rosa.9603@gmail.com>, Benedita Ferreira <ditaferre@gmail.com>, Clevio Nobrega <cdnobrega@ualg.pt>, Inês Torquato Afonso <inesta.28@gmail.com>, Luis Pereira de Almeida <lpereiradealmeida@gmail.com>, Rafael Costa <rafael.gm.costa@gmail.com>, Ricardo Reis <reisricardo12@hotmail.com>, Rui Nobre <rui.jorge.nobre@gmail.com>

Hi all,

I agree with the changes to the author list.

Kind regards,
João

On Fri, 12 Nov 2021, 12:32 Carina Santos Henriques, <cshenriques@cnc.uc.pt> wrote:

> Dear all,
>
> I do agree with the alterations regarding the new order of the authors' list.
>
> Best regards,
> Carina Henriques
>
> A sexta, 12/11/2021, 12:24, Carlos Matos <camatos@ualg.pt> escreveu:
>
>> I am in agreement with the changes.
>>
>> All the best,
>>
>> Carlos Matos
>>
>> David Brito <david.v.c.brito@gmail.com> escreveu em sex., 12/11/2021 às 12:18 :
>>
>>> Dear all,
>>>
>>> I agree with the above-mentioned changes.
>>>
>>> Best wishes,
>>>
>>> **David**
>>>
>>> On Fri, 12 Nov 2021 at 11:39, Clevio Nobrega <cdnobrega@ualg.pt> wrote:
>>>
>>>> Dear all,
>>>> As you know the paper "Autophagy in Spinocerebellar ataxia type 2, a dysregulated pathway, and a target for therapy" by Adriana was accepted in Cell death and disease. As several new experiments were made in this last round of reviews, we include two additional authors (Rafael and David) and changed the order list to include them and reflect the new work made. Now I need that you all confirm that you agree with this alteration.
>>>>
>>>> **So please reply by today to this email confirming that you agree with the changes! without this**, we cannot proceed with the publication procedure.
>>>>
>>>> Thank you in advance.
>>>>
>>>> Best
>>>>
>>>> **CLÉVIO** DAVID RODRIGUES **NÓBREGA**

Original authors: Adriana Marcelo,[1,2,3,4] Ana Rosa,[1] João Alves-Cruzeiro,[3] Benedita Ferreira,[1,4] Ricardo Reis,[1,4] Inês Afonso,[1,4] Carina Henriques,[3] Rui J. Nobre,[3] Carlos A. Matos,[1,4] Luís Pereira de Almeida,[3,5] Clévio Nóbrega [1,4,6*]

Final list:Adriana Marcelo,[1,2,3,4], Inês T. Afonso,[1] Ricardo Afonso-Reis,[1,4] David V.C. Brito,[1] Rafael G. Costa,[1,4] Ana Rosa,[1] João Alves-Cruzeiro,[3] Benedita Ferreira,[1,4] Carina Henriques,[3] Rui J. Nobre,[3] Carlos A. Matos,[1,4] Luís Pereira de Almeida,[3,5] Clévio Nóbrega [1,4,6*]

--
***Carlos A. Matos**, PhD*

Post-doctoral Associate Researcher
Algarve Biomedical Center - Research Institute (ABC-RI)
Faculty of Medicine and Biomedical Sciences
University of Algarve
Faro, Portugal

MNGT Molecular Neuroscience and Gene Therapy Lab
ABC Ri ALGARVE BIOMEDICAL CENTER RESEARCH INSTITUTE
UAlg FMCB UNIVERSIDADE DO ALGARVE FACULDADE DE MEDICINA E CIÊNCIAS BIOMÉDICAS
UAlg UNIVERSIDADE DO ALGARVE

Gmail **Clevio Nobrega <kl3vio@gmail.com>**

URGENTE - Paper Cell death and Disease - CDDIS-21-2430

David Brito <david.v.c.brito@gmail.com> 12 de novembro de 2021 às 12:18
Para: Clevio Nobrega <cdnobrega@ualg.pt>
Cc: Adriana do Vale <adrianamarcelo11@hotmail.com>, Inês Torquato Afonso <inesta.28@gmail.com>, Ricardo Reis <reisricardo12@hotmail.com>, Rafael Costa <rafael.gm.costa@gmail.com>, Benedita Ferreira <ditaferre@gmail.com>, João Cruzeiro <jc.joaocruzeiro@gmail.com>, cshenriques@cnc.uc.pt, Carlos Adriano Albuquerque Andrade de Matos <camatos@ualg.pt>, Luis Pereira de Almeida <lpereiradealmeida@gmail.com>, Rui Nobre <rui.jorge.nobre@gmail.com>, ANA RITA TIAGO ROSA <a52713@ualg.pt>, "Ana (Solo) Rosa" <ana.rosa.9603@gmail.com>

Dear all,

I agree with the above-mentioned changes.

Best wishes,

David

On Fri, 12 Nov 2021 at 11:39, Clevio Nobrega <cdnobrega@ualg.pt> wrote:

> Dear all,
> As you know the paper "Autophagy in Spinocerebellar ataxia type 2, a dysregulated pathway, and a target for therapy" by Adriana was accepted in Cell death and disease. As several new experiments were made in this last round of reviews, we include two additional authors (Rafael and David) and changed the order list to include them and reflect the new work made. Now I need that you all confirm that you agree with this alteration.
>
> **So please reply by today to this email confirming that you agree with the changes! without this**, we cannot proceed with the publication procedure.
>
> Thank you in advance.
>
> Best
>
> **CLÉVIO** DAVID RODRIGUES **NÓBREGA**
>
> Original authors: Adriana Marcelo,[1,2,3,4] Ana Rosa,[1] João Alves-Cruzeiro,[3] Benedita Ferreira,[1,4] Ricardo Reis,[1,4] Inês Afonso,[1,4] Carina Henriques,[3] Rui J. Nobre,[3] Carlos A. Matos,[1,4] Luís Pereira de Almeida,[3,5] Clévio Nóbrega [1,4,6*]
>
> Final list:Adriana Marcelo,[1,2,3,4], Inês T. Afonso,[1] Ricardo Afonso-Reis,[1,4] David V.C. Brito,[1] Rafael G. Costa,[1,4] Ana Rosa,[1] João Alves-Cruzeiro,[3] Benedita Ferreira,[1,4] Carina Henriques,[3] Rui J. Nobre,[3] Carlos A. Matos,[1,4] Luís Pereira de Almeida,[3,5] Clévio Nóbrega [1,4,6*]

Gmail **Clevio Nobrega <kl3vio@gmail.com>**

URGENTE - Paper Cell death and Disease - CDDIS-21-2430

Benedita Ferreira <ditaferre@gmail.com> 12 de novembro de 2021 às 15:55
Para: Clevio Nobrega <cdnobrega@ualg.pt>
Cc: Adriana do Vale <adrianamarcelo11@hotmail.com>, Inês Torquato Afonso <inesta.28@gmail.com>, Ricardo Reis <reisricardo12@hotmail.com>, David Brito <david.v.c.brito@gmail.com>, Rafael Costa <rafael.gm.costa@gmail.com>, Benedita Ferreira <ditaferre@gmail.com>, João Cruzeiro <jc.joaocruzeiro@gmail.com>, cshenriques@cnc.uc.pt, Carlos Adriano Albuquerque Andrade de Matos <camatos@ualg.pt>, Luis Pereira de Almeida <lpereiradealmeida@gmail.com>, Rui Nobre <rui.jorge.nobre@gmail.com>, ANA RITA TIAGO ROSA <a52713@ualg.pt>, "Ana (Solo) Rosa" <ana.rosa.9603@gmail.com>

Dear all,

Congratulations!!
I do agree with the alterations regarding the order of the authors' list.

Best regards,

Benedita Ferreira

Clevio Nobrega <cdnobrega@ualg.pt> escreveu no dia sexta, 12/11/2021 à(s) 11:39:

> Dear all,
> As you know the paper "Autophagy in Spinocerebellar ataxia type 2, a dysregulated pathway, and a target for therapy" by Adriana was accepted in Cell death and disease. As several new experiments were made in this last round of reviews, we include two additional authors (Rafael and David) and changed the order list to include them and reflect the new work made. Now I need that you all confirm that you agree with this alteration.
>
> **So please reply by today to this email confirming that you agree with the changes! without this**, we cannot proceed with the publication procedure.
>
> Thank you in advance.
>
> Best
>
> **CLÉVIO** DAVID RODRIGUES **NÓBREGA**
>
> Original authors: Adriana Marcelo,[1,2,3,4] Ana Rosa,[1] João Alves-Cruzeiro,[3] Benedita Ferreira,[1,4] Ricardo Reis,[1,4] Inês Afonso,[1,4] Carina Henriques,[3] Rui J. Nobre,[3] Carlos A. Matos,[1,4] Luís Pereira de Almeida,[3,5] Clévio Nóbrega [1,4,6*]
>
> Final list:Adriana Marcelo,[1,2,3,4], Inês T. Afonso,[1] Ricardo Afonso-Reis,[1,4] David V.C. Brito,[1] Rafael G. Costa,[1,4] Ana Rosa,[1] João Alves-Cruzeiro,[3] Benedita Ferreira,[1,4] Carina Henriques,[3] Rui J. Nobre,[3] Carlos A. Matos,[1,4] Luís Pereira de Almeida,[3,5] Clévio Nóbrega [1,4,6*]

--

10 ANOS MEDICINA UNIVERSIDADE DO ALGARVE
EXCELÊNCIA . INOVAÇÃO . HUMANISMO

Gmail **Clevio Nobrega <kl3vio@gmail.com>**

URGENTE - Paper Cell death and Disease - CDDIS-21-2430

Inês Afonso <inesta.28@gmail.com> 12 de novembro de 2021 às 11:54
Para: Clevio Nobrega <cdnobrega@ualg.pt>
Cc: Adriana do Vale <adrianamarcelo11@hotmail.com>, Ricardo Reis <reisricardo12@hotmail.com>, David Brito <david.v.c.brito@gmail.com>, Rafael Costa <rafael.gm.costa@gmail.com>, Benedita Ferreira <ditaferre@gmail.com>, João Cruzeiro <jc.joaocruzeiro@gmail.com>, cshenriques@cnc.uc.pt, Carlos Adriano Albuquerque Andrade de Matos <camatos@ualg.pt>, Luis Pereira de Almeida <lpereiradealmeida@gmail.com>, Rui Nobre <rui.jorge.nobre@gmail.com>, ANA RITA TIAGO ROSA <a52713@ualg.pt>, "Ana (Solo) Rosa" <ana.rosa.9603@gmail.com>

Dear all,

I agree with the alterations.

Best wishes,

Inês Afonso

Clevio Nobrega <cdnobrega@ualg.pt> escreveu no dia sexta, 12/11/2021 à(s) 11:39:

> Dear all,
> As you know the paper "Autophagy in Spinocerebellar ataxia type 2, a dysregulated pathway, and a target for therapy" by Adriana was accepted in Cell death and disease. As several new experiments were made in this last round of reviews, we include two additional authors (Rafael and David) and changed the order list to include them and reflect the new work made. Now I need that you all confirm that you agree with this alteration.
>
> **So please reply by today to this email confirming that you agree with the changes! without this**, we cannot proceed with the publication procedure.
>
> Thank you in advance.
>
> Best
>
> **CLÉVIO** DAVID RODRIGUES **NÓBREGA**
>
> Original authors: Adriana Marcelo,[1,2,3,4] Ana Rosa,[1] João Alves-Cruzeiro,[3] Benedita Ferreira,[1,4] Ricardo Reis,[1,4] Inês Afonso,[1,4] Carina Henriques,[3] Rui J. Nobre,[3] Carlos A. Matos,[1,4] Luís Pereira de Almeida,[3,5] Clévio Nóbrega [1,4,6*]
>
> Final list:Adriana Marcelo,[1,2,3,4], Inês T. Afonso,[1] Ricardo Afonso-Reis,[1,4] David V.C. Brito,[1] Rafael G. Costa,[1,4] Ana Rosa,[1] João Alves-Cruzeiro,[3] Benedita Ferreira,[1,4] Carina Henriques,[3] Rui J. Nobre,[3] Carlos A. Matos,[1,4] Luís Pereira de Almeida,[3,5] Clévio Nóbrega [1,4,6*]

Gmail **Clevio Nobrega <kl3vio@gmail.com>**

URGENTE - Paper Cell death and Disease - CDDIS-21-2430

Luis Pereira de Almeida <luispa@cnc.uc.pt> 12 de novembro de 2021 às 12:00
Para: Rafael Costa <rafael.gm.costa@gmail.com>
Cc: Clevio Nobrega <cdnobrega@ualg.pt>, Adriana do Vale <adrianamarcelo11@hotmail.com>, Inês Torquato Afonso <inesta.28@gmail.com>, Ricardo Reis <reisricardo12@hotmail.com>, David Brito <david.v.c.brito@gmail.com>, Benedita Ferreira <ditaferre@gmail.com>, João Cruzeiro <jc.joaocruzeiro@gmail.com>, cshenriques@cnc.uc.pt, Carlos Adriano Albuquerque Andrade de Matos <camatos@ualg.pt>, Rui Nobre <rui.jorge.nobre@gmail.com>, ANA RITA TIAGO ROSA <a52713@ualg.pt>, "Ana (Solo) Rosa" <ana.rosa.9603@gmail.com>

Dear all,
I agree with the changes in the list of authors.
All the best,
Luis Almeida

On Fri, 12 Nov 2021 at 11:56, Rafael Costa <rafael.gm.costa@gmail.com> wrote:

> Dear all,
>
> I do agree with the alterations regarding the order of the authors' list.
>
> Best regards,
> ***Rafael G. Costa***
> *------*
> *MSc in Biomedical Sciences (student)*
> *------*
> *Molecular Neuroscience and Gene Therapy group*
> *Algarve Biomedical Center Research Institute (ABC-RI)*
> *Faculty of Medicine and Biomedical Sciences*
> *Universidade do Algarve*
> *Gambelas campus*
> *8005-139 Faro, Portugal*
>
> On Fri, Nov 12, 2021 at 11:39 AM Clevio Nobrega <cdnobrega@ualg.pt> wrote:
>
>> Dear all,
>> As you know the paper "Autophagy in Spinocerebellar ataxia type 2, a dysregulated pathway, and a target for therapy" by Adriana was accepted in Cell death and disease. As several new experiments were made in this last round of reviews, we include two additional authors (Rafael and David) and changed the order list to include them and reflect the new work made. Now I need that you all confirm that you agree with this alteration.
>>
>> **So please reply by today to this email confirming that you agree with the changes! without this**, we cannot proceed with the publication procedure.
>>
>> Thank you in advance.
>>
>> Best
>>
>> **CLÉVIO** DAVID RODRIGUES **NÓBREGA**
>>
>> Original authors: Adriana Marcelo,[1,2,3,4] Ana Rosa,[1] João Alves-Cruzeiro,[3] Benedita Ferreira,[1,4] Ricardo Reis,[1,4] Inês Afonso,[1,4] Carina Henriques,[3] Rui J. Nobre,[3] Carlos A. Matos,[1,4] Luís Pereira de Almeida,[3,5] Clévio Nóbrega [1,4,6*]
>>
>> Final list:Adriana Marcelo,[1,2,3,4], Inês T. Afonso,[1] Ricardo Afonso-Reis,[1,4] David V.C. Brito,[1] Rafael G. Costa,[1,4] Ana Rosa,[1] João Alves-Cruzeiro,[3] Benedita Ferreira,[1,4] Carina

Henriques,[3] Rui J. Nobre,[3] Carlos A. Matos,[1,4] Luís Pereira de Almeida,[3,5] Clévio Nóbrega [1,4,6*]

--

Luis Pereira de Almeida
CNC - Center for Neuroscience and Cell Biology
CIBB - Center for Innovative Biomedicine and Biotechnology
University of Coimbra
Rua Larga - Pólo 1
3004-504 Coimbra - Portugal
https://www.cibb.uc.pt/research/innovative-therapies/vectors-gene-cell-therapy/

and Faculty of Pharmacy, University of Coimbra
Pólo das Ciências da Saúde, Azinhaga de Santa Comba - Pólo 3
3000-548 Coimbra - Portugal
E-mail: luispa@cnc.uc.pt   Tel: +351966337482

Gmail

Clevio Nobrega <kl3vio@gmail.com>

URGENTE - Paper Cell death and Disease - CDDIS-21-2430

Rafael Costa <rafael.gm.costa@gmail.com> 12 de novembro de 2021 às 11:56
Para: Clevio Nobrega <cdnobrega@ualg.pt>
Cc: Adriana do Vale <adrianamarcelo11@hotmail.com>, Inês Torquato Afonso <inesta.28@gmail.com>, Ricardo Reis <reisricardo12@hotmail.com>, David Brito <david.v.c.brito@gmail.com>, Benedita Ferreira <ditaferre@gmail.com>, João Cruzeiro <jc.joaocruzeiro@gmail.com>, cshenriques@cnc.uc.pt, Carlos Adriano Albuquerque Andrade de Matos <camatos@ualg.pt>, Luis Pereira de Almeida <lpereiradealmeida@gmail.com>, Rui Nobre <rui.jorge.nobre@gmail.com>, ANA RITA TIAGO ROSA <a52713@ualg.pt>, "Ana (Solo) Rosa" <ana.rosa.9603@gmail.com>

Dear all,

I do agree with the alterations regarding the order of the authors' list.

Best regards,
Rafael G. Costa

MSc in Biomedical Sciences (student)

Molecular Neuroscience and Gene Therapy group
Algarve Biomedical Center Research Institute (ABC-RI)
Faculty of Medicine and Biomedical Sciences
Universidade do Algarve
Gambelas campus
8005-139 Faro, Portugal

MNGT Molecular Neuroscience and Gene Therapy Lab | ABC Ri ALGARVE BIOMEDICAL CENTER RESEARCH INSTITUTE | UAlg FMCB UNIVERSIDADE DO ALGARVE FACULDADE DE MEDICINA E CIÊNCIAS BIOMÉDICAS | UAlg UNIVERSIDADE DO ALGARVE

On Fri, Nov 12, 2021 at 11:39 AM Clevio Nobrega <cdnobrega@ualg.pt> wrote:

> Dear all,
> As you know the paper "Autophagy in Spinocerebellar ataxia type 2, a dysregulated pathway, and a target for therapy" by Adriana was accepted in Cell death and disease. As several new experiments were made in this last round of reviews, we include two additional authors (Rafael and David) and changed the order list to include them and reflect the new work made. Now I need that you all confirm that you agree with this alteration.
>
> **So please reply by today to this email confirming that you agree with the changes! without this**, we cannot proceed with the publication procedure.
>
> Thank you in advance.
>
> Best
>
> **CLÉVIO** DAVID RODRIGUES **NÓBREGA**
>
> Original authors: Adriana Marcelo,[1,2,3,4] Ana Rosa,[1] João Alves-Cruzeiro,[3] Benedita Ferreira,[1,4] Ricardo Reis,[1,4] Inês Afonso,[1,4] Carina Henriques,[3] Rui J. Nobre,[3] Carlos A. Matos,[1,4] Luís Pereira de Almeida,[3,5] Clévio Nóbrega [1,4,6*]
>
> Final list:Adriana Marcelo,[1,2,3,4], Inês T. Afonso,[1] Ricardo Afonso-Reis,[1,4] David V.C. Brito,[1] Rafael G. Costa,[1,4] Ana Rosa,[1] João Alves-Cruzeiro,[3] Benedita Ferreira,[1,4] Carina Henriques,[3] Rui J. Nobre,[3] Carlos A. Matos,[1,4] Luís Pereira de Almeida,[3,5] Clévio Nóbrega [1,4,6*]

Gmail **Clevio Nobrega <kl3vio@gmail.com>**

URGENTE - Paper Cell death and Disease - CDDIS-21-2430

---

ricardo reis <reisricardo12@hotmail.com> 12 de novembro de 2021 às 11:57
Para: Inês Afonso <inesta.28@gmail.com>, Clevio Nobrega <cdnobrega@ualg.pt>
Cc: Adriana do Vale <adrianamarcelo11@hotmail.com>, David Brito <david.v.c.brito@gmail.com>, Rafael Costa <rafael.gm.costa@gmail.com>, Benedita Ferreira <ditaferre@gmail.com>, João Cruzeiro <jc.joaocruzeiro@gmail.com>, "cshenriques@cnc.uc.pt" <cshenriques@cnc.uc.pt>, Carlos Adriano Albuquerque Andrade de Matos <camatos@ualg.pt>, Luis Pereira de Almeida <lpereiradealmeida@gmail.com>, Rui Nobre <rui.jorge.nobre@gmail.com>, ANA RITA TIAGO ROSA <a52713@ualg.pt>, "Ana (Solo) Rosa" <ana.rosa.9603@gmail.com>

I hereby declare that I agree with the alterations performed in the final list of authors of the paper "Autophagy in Spinocerebellar ataxia type 2, a dysregulated pathway, and a target for therapy".

Best regards

Ricardo Afonso-Reis

De: Inês Afonso <inesta.28@gmail.com>
Enviado: 12 de novembro de 2021 11:54
Para: Clevio Nobrega <cdnobrega@ualg.pt>
Cc: Adriana do Vale <adrianamarcelo11@hotmail.com>; Ricardo Reis <reisricardo12@hotmail.com>; David Brito <david.v.c.brito@gmail.com>; Rafael Costa <rafael.gm.costa@gmail.com>; Benedita Ferreira <ditaferre@gmail.com>; João Cruzeiro <jc.joaocruzeiro@gmail.com>; cshenriques@cnc.uc.pt <cshenriques@cnc.uc.pt>; Carlos Adriano Albuquerque Andrade de Matos <camatos@ualg.pt>; Luis Pereira de Almeida <lpereiradealmeida@gmail.com>; Rui Nobre <rui.jorge.nobre@gmail.com>; ANA RITA TIAGO ROSA <a52713@ualg.pt>; Ana (Solo) Rosa <ana.rosa.9603@gmail.com>
Assunto: Re: URGENTE - Paper Cell death and Disease - CDDIS-21-2430

Dear all,

I agree with the alterations.

Best wishes,

Inês Afonso

Clevio Nobrega <cdnobrega@ualg.pt> escreveu no dia sexta, 12/11/2021 à(s) 11:39:

> Dear all,
> As you know the paper "Autophagy in Spinocerebellar ataxia type 2, a dysregulated pathway, and a target for therapy" by Adriana was accepted in Cell death and disease. As several new experiments were made in this last round of reviews, we include two additional authors (Rafael and David) and changed the order list to include them and reflect the new work made. Now I need that you all confirm that you agree with this alteration.
>
> **So please reply by today to this email confirming that you agree with the changes! without this**, we cannot proceed with the publication procedure.
>
> Thank you in advance.
>
> Best
>
> **CLÉVIO** DAVID RODRIGUES **NÓBREGA**
>
> Original authors: Adriana Marcelo,[1,2,3,4] Ana Rosa,[1] João Alves-Cruzeiro,[3] Benedita Ferreira,[1,4] Ricardo Reis,[1,4] Inês Afonso,[1,4] Carina Henriques,[3] Rui J. Nobre,[3] Carlos A. Matos,[1,4] Luís Pereira de Almeida,[3,5] Clévio Nóbrega [1,4,6*]

Final list:Adriana Marcelo,[1,2,3,4], Inês T. Afonso,[1] Ricardo Afonso-Reis,[1,4] David V.C. Brito,[1] Rafael G. Costa,[1,4] Ana Rosa,[1] João Alves-Cruzeiro,[3] Benedita Ferreira,[1,4] Carina Henriques,[3] Rui J. Nobre,[3] Carlos A. Matos,[1,4] Luís Pereira de Almeida,[3,5] Clévio Nóbrega [1,4,6*]

Gmail

Clevio Nobrega <kl3vio@gmail.com>

URGENTE - Paper Cell death and Disease - CDDIS-21-2430

Rui Nobre <rui.nobre@cnc.uc.pt> 12 de novembro de 2021 às 12:28
Para: Clevio Nobrega <cdnobrega@ualg.pt>
Cc: David Brito <david.v.c.brito@gmail.com>, ANA RITA TIAGO ROSA <a52713@ualg.pt>, Adriana do Vale <adrianamarcelo11@hotmail.com>, "Ana (Solo) Rosa" <ana.rosa.9603@gmail.com>, Benedita Ferreira <ditaferre@gmail.com>, Inês Torquato Afonso <inesta.28@gmail.com>, João Cruzeiro <jc.joaocruzeiro@gmail.com>, Luis Pereira de Almeida <lpereiradealmeida@gmail.com>, Rafael Costa <rafael.gm.costa@gmail.com>, Ricardo Reis <reisricardo12@hotmail.com>, cshenriques@cnc.uc.pt, Rui Nobre <rui.jorge.nobre@gmail.com>, Carlos Matos <camatos@ualg.pt>, Carina Henriques <chenriques4657@gmail.com>

Dear all,

I do agree with the alterations regarding the new order of the authors' list.

Best regards,
RUI NOBRE

--
Rui Jorge Nobre
. **Institute for Interdisciplinary Research @ UC**

. **ViraVector- Viral Vectors for Gene Transfer Core facility @ UC**

. **Center for Neuroscience and Cell Biology (CNC),**

University of Coimbra (UC), 3004-517 Coimbra, Portugal
e-mail: rui.nobre@cnc.uc.pt

On Fri, Nov 12, 2021 at 12:25 PM Carlos Matos <camatos@ualg.pt> wrote:

> I am in agreement with the changes.
>
> All the best,
>
> Carlos Matos
>
> David Brito <david.v.c.brito@gmail.com> escreveu em sex., 12/11/2021 às 12:18 :
>
>> Dear all,
>>
>> I agree with the above-mentioned changes.
>>
>> Best wishes,
>>
>> **David**
>>
>> On Fri, 12 Nov 2021 at 11:39, Clevio Nobrega <cdnobrega@ualg.pt> wrote:
>>
>>> Dear all,
>>> As you know the paper "Autophagy in Spinocerebellar ataxia type 2, a dysregulated pathway, and a target for therapy" by Adriana was accepted in Cell death and disease. As several new experiments were made in this last round of reviews, we include two additional authors (Rafael and David) and changed the order list to include them and reflect the new work made. Now I need that you all confirm that you agree with this alteration.
>>>
>>> **So please reply by today to this email confirming that you agree with the changes! without this**, we cannot proceed with the publication procedure.
>>>
>>> Thank you in advance.
>>>
>>> Best
>>>
>>> **CLÉVIO** DAVID RODRIGUES **NÓBREGA**

Original authors: Adriana Marcelo,[1,2,3,4] Ana Rosa,[1] João Alves-Cruzeiro,[3] Benedita Ferreira,[1,4] Ricardo Reis,[1,4] Inês Afonso,[1,4] Carina Henriques,[3] Rui J. Nobre,[3] Carlos A. Matos,[1,4] Luís Pereira de Almeida,[3,5] Clévio Nóbrega [1,4,6*]

Final list:Adriana Marcelo,[1,2,3,4], Inês T. Afonso,[1] Ricardo Afonso-Reis,[1,4] David V.C. Brito,[1] Rafael G. Costa,[1,4] Ana Rosa,[1] João Alves-Cruzeiro,[3] Benedita Ferreira,[1,4] Carina Henriques,[3] Rui J. Nobre,[3] Carlos A. Matos,[1,4] Luís Pereira de Almeida,[3,5] Clévio Nóbrega [1,4,6*]

--

***Carlos A. Matos**, PhD*

Post-doctoral Associate Researcher
Algarve Biomedical Center - Research Institute (ABC-RI)
Faculty of Medicine and Biomedical Sciences
University of Algarve
Faro, Portugal
